# Transmembrane serine protease 2 (TMPRSS2) proteolytically activates the epithelial sodium channel (ENaC) by cleaving the channel’s γ-subunit

**DOI:** 10.1016/j.jbc.2022.102004

**Published:** 2022-04-30

**Authors:** Florian Sure, Marko Bertog, Sara Afonso, Alexei Diakov, Ralf Rinke, M. Gregor Madej, Sabine Wittmann, Thomas Gramberg, Christoph Korbmacher, Alexandr V. Ilyaskin

**Affiliations:** 1 Friedrich-Alexander-Universität Erlangen-Nürnberg, Institute of Cellular and Molecular Physiology, Erlangen, Germany; 2 Department of Biophysics II/Structural Biology, University of Regensburg, Regensburg, Germany; 3 Friedrich-Alexander-Universität Erlangen-Nürnberg, Universitätsklinikum Erlangen, Institute of Clinical and Molecular Virology, Erlangen, Germany

**Keywords:** epithelial sodium channel, TMPRSS2, proteolytic channel activation, two-electrode voltage clamp, electrophysiology, oocyte, homology modeling, serine protease, epithelial cell, H441 cell line, ami, amiloride, ASL, apical surface layer, Boc–QAR–AMC, butyloxycarbonyl–Gln-Ala-Arg–7-amino-4-methylcoumarin, CAP1, channel-activating protease 1, Cas9, CRISPR-associated protein 9, cDNA, complementary DNA, cRNA, complementary RNA, ENaC, epithelial sodium channel, GRIP, gating relief of inhibition by proteolysis, HA, hemagglutinin, luc, firefly luciferase, MTSET, 2-(trimethylammonium)ethyl methanethiosulfonate bromide, *P*_O_, open probability, sgRNA, single-guide RNA, TMPRSS2, transmembrane serine protease 2, TMPRSS4, transmembrane serine protease 4, γ-11, key inhibitory amino acid sequence of γ-ENaC

## Abstract

The epithelial sodium channel (ENaC) is a heterotrimer consisting of α-, β-, and γ-subunits. Channel activation requires proteolytic release of inhibitory tracts from the extracellular domains of α-ENaC and γ-ENaC; however, the proteases involved in the removal of the γ-inhibitory tract remain unclear. In several epithelial tissues, ENaC is coexpressed with the transmembrane serine protease 2 (TMPRSS2). Here, we explored the effect of human TMPRSS2 on human αβγ-ENaC heterologously expressed in *Xenopus laevis* oocytes. We found that coexpression of TMPRSS2 stimulated ENaC-mediated whole-cell currents by approximately threefold, likely because of an increase in average channel open probability. Furthermore, TMPRSS2-dependent ENaC stimulation was not observed using a catalytically inactive TMPRSS2 mutant and was associated with fully cleaved γ-ENaC in the intracellular and cell surface protein fractions. This stimulatory effect of TMPRSS2 on ENaC was partially preserved when inhibiting its proteolytic activity at the cell surface using aprotinin but was abolished when the γ-inhibitory tract remained attached to its binding site following introduction of two cysteine residues (S155C–Q426C) to form a disulfide bridge. In addition, computer simulations and site-directed mutagenesis experiments indicated that TMPRSS2 can cleave γ-ENaC at sites both proximal and distal to the γ-inhibitory tract. This suggests a dual role of TMPRSS2 in the proteolytic release of the γ-inhibitory tract. Finally, we demonstrated that TMPRSS2 knockdown in cultured human airway epithelial cells (H441) reduced baseline proteolytic activation of endogenously expressed ENaC. Thus, we conclude that TMPRSS2 is likely to contribute to proteolytic ENaC activation in epithelial tissues *in vivo*.

The epithelial sodium channel (ENaC) belongs to the ENaC/degenerin family of ion channels (1). As rate-limiting pathway for apical sodium entry, ENaC is critically involved in transepithelial sodium absorption in the aldosterone-sensitive distal nephron, distal colon, respiratory epithelia, as well as sweat and salivary ducts (2, 3, 4, 5, 6). ENaC is a heterotrimer consisting of three homologous subunits (α, β, and γ) (7, 8). In several vertebrate species including humans, an additional δ-subunit exists that can functionally replace the α-subunit in heterologous expression systems, thereby altering channel function and regulation (9, 10, 11). A unique feature of ENaC is its proteolytic activation (12). Proteases stimulate ENaC by cleaving specific sites in the extracellular loops of its α-subunit and γ-subunit but not of its β-subunit (13, 14). Cleavage results in the release of inhibitory tracts and probably activates the channel by changing its conformation (14, 15). Recently published cryo-EM structural data of ENaC indicate that specific binding sites are present to allow a close interaction of the key inhibitory amino acid sequences of the α-inhibitory and γ-inhibitory tracts with their respective subunits. These binding sites are formed by parts of the extracellular finger, thumb, and GRIP (gating relief of inhibition by proteolysis) domains present in each subunit (7, 8). These findings are in a good agreement with functional data obtained using synthetic inhibitory peptides corresponding to these key inhibitory sequences (16, 17). As long as these binding sites are occupied by the inhibitory tracts or by exogenously applied synthetic inhibitory peptides (9, 18, 19), the channel tends to be inactive. In contrast, proteolytic removal of the inhibitory tracts results in channel activation. For complete channel activation, release of the γ-inhibitory tract by dual cleavage of the channel’s γ-subunit is thought to be the most important mechanism (20). The currently accepted paradigm of proteolytic ENaC activation is mainly based on results obtained in heterologous expression systems. According to this, three cleavage sites (two in α-ENaC and one in γ-ENaC) are targeted by furin and/or related furin-like proprotein convertases during channel maturation in the intracellular biosynthetic pathway (14, 21). The pivotal final step of proteolytic ENaC activation is assumed to take place at the plasma membrane where γ-ENaC is cleaved by membrane-anchored and/or extracellular proteases in a region distal to the furin site (14, 22, 23, 24).

Relevant proteases involved in proteolytic ENaC activation under physiological conditions *in vivo* remain elusive (25). Various serine proteases, including trypsin (26, 27, 28), chymotrypsin (24, 26), plasmin (29, 30, 31), kallikrein (32, 33), elastase (34, 35, 36, 37), and trypsin IV (28), but also metalloproteases (38) and the cysteine protease cathepsin S (39), have been shown to cleave γ-ENaC and activate the channel when exogenously applied from the extracellular side. Interestingly, multiple cleavage sites with distinct protease preferences have been identified, in particular for the distal cleavage event in γ-ENaC (22, 24, 28, 31, 40, 41, 42). Thus, the size of the released γ-inhibitory tract of about ∼40 amino acid residues may slightly vary depending on the cleavage site preferentially targeted by the responsible protease.

Importantly, in expression systems, ENaC can be fully activated by coexpressing membrane-anchored serine proteases (43, 44, 45, 46). The family of membrane-anchored serine proteases consists of 20 members identified in human to date (47, 48, 49). They share a conserved extracellular catalytic domain and are anchored directly to the plasma membrane *via* a glycosylphosphatidylinositol anchor or a transmembrane domain. Prostasin (PRSS8) was the first membrane-anchored serine protease demonstrated to activate ENaC in coexpression experiments and was therefore named channel-activating protease 1 (CAP1) (43, 50, 51, 52). There is evidence from genetically modified mouse models that PRSS8 is involved in ENaC regulation in distal colon and alveolar epithelium (53, 54). However, it remains debatable whether PRSS8 contributes to proteolytic ENaC regulation in the kidney and other ENaC-expressing tissues (25). Interestingly, studies in heterologous expression systems and mouse models indicate that PRSS8 does not require its proteolytic activity to activate ENaC. Instead, PRSS8 may activate ENaC by recruiting additional endogenous proteases, which remain to be identified (22, 45, 55, 56). In contrast, the stimulatory effect of transmembrane serine protease 4 (TMPRSS4, CAP2) and matriptase (CAP3) on ENaC has been shown to depend on their proteolytic activity (41, 45, 46).

Interestingly, conflicting data have been reported regarding the effect of the TMPRSS2 (or epitheliasin) on ENaC function. An initial study found that coexpression of human TMPRSS2 with rat ENaC in *Xenopus laevis* oocytes dramatically reduced ENaC-mediated currents and ENaC protein expression (57). This finding led to the concept that TMPRSS2 is an inhibitory protease for ENaC (58). In contrast, a stimulatory effect of coexpressed human TMPRSS2 on rat ENaC has been reported (46). Thus, the role of TMPRSS2 in proteolytic ENaC regulation remains to be clarified. TMPRSS2 is a trypsin-like serine protease in which the catalytic triad is formed by conserved histidine, aspartate, and serine residues (59, 60, 61). TMPRSS2 has a substrate specificity similar to that of trypsin, cleaving after positively charged arginine or lysine residues at the P1 position (62). Thus, TMPRSS2 and trypsin may activate ENaC in a similar manner. Moreover, TMPRSS2 was shown to be highly expressed in renal distal tubule, airway epithelial cells, and distal colon (46, 57, 60, 61, 63, 64, 65), where TMPRSS2 may colocalize and functionally interact with ENaC.

In this study, we used the *X. laevis* oocyte expression system to provide evidence for proteolytic ENaC activation by TMPRSS2. Moreover, we identified putative TMPRSS2 cleavage sites in the channel’s γ-subunit. To investigate a possible regulatory effect of TMPRSS2 on ENaC function in polarized epithelial cells, we also studied the effect of TMPRSS2 knockdown on ENaC-mediated transepithelial sodium transport in H441 human distal airway epithelial cells.

## Results

### Stimulatory effect of TMPRSS2 coexpression on ENaC-mediated amiloride-sensitive whole-cell currents depends on the proteolytic activity of TMPRSS2

**Figure 1:**
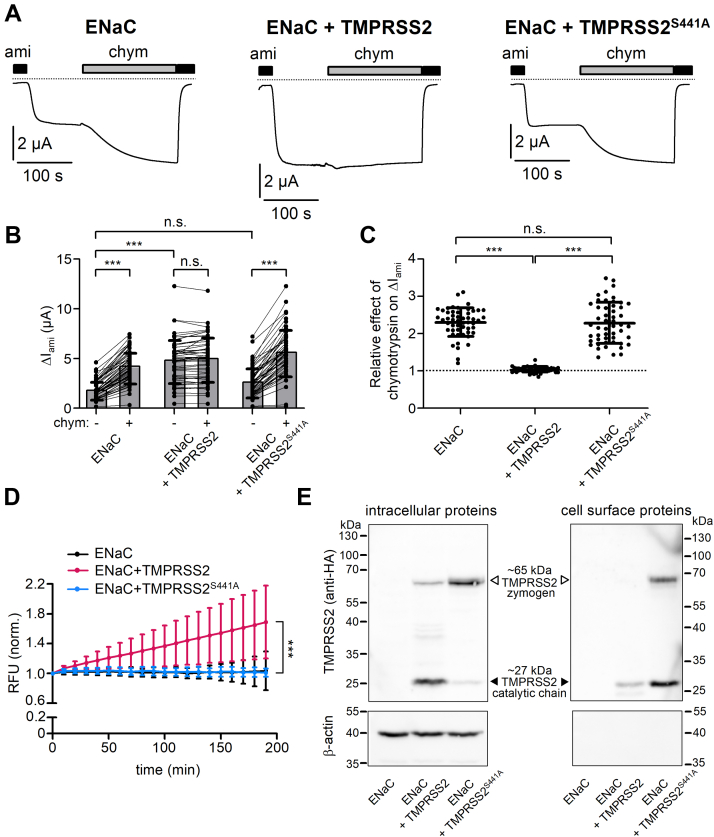
**Stimulatory effect of TMPRSS2 on ENaC requires proteolytic activity of TMPRSS2.***A*, representative whole-cell current traces are shown for oocytes expressing human wildtype αβγ-ENaC alone (*left trace*, ENaC) or coexpressing αβγ-ENaC with human wildtype TMPRSS2 (*middle trace*, ENaC + TMPRSS2) or catalytically inactive TMPRSS2 (*right trace*, ENaC + TMPRSS2^S441A^). HA tag epitope was attached to the C terminus of TMPRSS2. HA tag neither disturbed the proteolytic activity of TMPRSS2 nor affected the stimulatory effect of TMPRSS2 on ENaC (Fig. S1). Amiloride (ami, 2 μM) and chymotrypsin (chym, 2 μg/ml) were present in the bath solution as indicated by *black* and *gray bars*, respectively. *Dashed lines* indicate zero current level. *B*, ENaC-mediated ami-sensitive whole-cell currents (ΔI_ami_) were determined from similar experiments as shown in (*A*) by subtracting the baseline current in the presence of ami from the current level reached in its absence before (−) or after (+) chym application. *Lines* connect data points obtained in an individual oocyte. Mean ± SD and data points for individual oocytes are shown; ∗∗∗*p* < 0.001; ns, Kruskal–Wallis with Dunn’s post hoc test (51 ≤ n ≤ 52, N = 5). *C*, relative stimulatory effect of chym on ΔI_ami_ summarized from data shown in (*B*). *Dashed line* indicates normalized ΔI_ami_ value of one (no effect). Mean ± SD and data points for individual oocytes are shown; ∗∗∗*p* < 0.001; ns, one-way ANOVA with Bonferroni post hoc test. *D*, in parallel experiments to those shown in (*A–C*), trypsin-like proteolytic activity at the cell surface was detected in the same batches of oocytes. Progress curves of proteolytic activity (RFU = relative fluorescent unit; mean ± SD) are shown. In each individual recording RFU values were normalized to the initial RFU value at the beginning of the measurement. ∗∗∗*p* < 0.001; Kruskal–Wallis with Dunn’s post hoc test (at the time point 190 min; 41≤ n ≤ 46, N = 5). *E*, representative western blots showing intracellular (*left upper panel*) or cell surface (*right upper panel*) expression of HA-tagged wildtype TMPRSS2 or mutant TMPRSS2^S441A^ in oocytes from one batch. No specific signal was detected with the anti-HA antibody in oocytes expressing ENaC alone. TMPRSS2 zymogen (∼65 kDa) and TMPRSS2 in its activated cleaved form (catalytic chain, ∼27 kDa) are indicated by *open* and *filled arrowheads*, respectively. To validate separation of cell surface proteins from intracellular proteins, blots were stripped and reprobed using an antibody against β-actin (*lower panels*). Similar results were obtained in three additional repeats (n = 4). ENaC, epithelial sodium channel; HA, hemagglutinin; ns, not significant; TMPRSS2, transmembrane serine protease 2.

To investigate whether human TMPRSS2 can modify ENaC function, we expressed human αβγ-ENaC in *X. laevis* oocytes with or without human TMPRSS2. ENaC function was assessed by measuring amiloride-sensitive inward currents (ΔI_ami_) using the two-electrode voltage clamp technique. Figure 1*A* shows representative continuous whole-cell current traces recorded at a holding potential of −60 mV in individual oocytes expressing ENaC alone (*left panel*), coexpressing ENaC and TMPRSS2 (*middle panel*), or coexpressing ENaC and a catalytically inactive mutant TMPRSS2 (TMPRSS2^S441A^; *right panel*). Results from similar experiments are summarized in Figure 1*B*. Recordings were started in the presence of 2 μM amiloride, which in this concentration is known to reversibly inhibit human ENaC heterologously expressed in oocytes by >90% (9, 66). Washout of amiloride revealed an ENaC-mediated Na^+^ inward current component.

It is well established that in the oocyte expression system a sizeable fraction of ENaC is not fully cleaved when it reaches the cell surface. These latter channels can be activated by extracellular application of prototypical serine proteases like trypsin or chymotrypsin (12, 14). Our finding, that in oocytes expressing ENaC alone (Fig. 1*A*, *left panel*) application of chymotrypsin (2 μg/ml) further increased the inward current by ∼2.5-fold, is in good agreement with this concept (Fig. 1, *B* and *C*). Reapplication of amiloride at the end of the measurement returned the current to its initial level. This confirms that the inward current stimulated by chymotrypsin is mediated by ENaC. Importantly, in oocytes coexpressing ENaC and TMPRSS2 (Fig. 1*A*, *middle panel*), baseline ΔI_ami_ was ∼2.5-fold larger than that in oocytes expressing ENaC alone (Fig. 1*B*). Moreover, in oocytes coexpressing ENaC and TMPRSS2, application of chymotrypsin had no additional stimulatory effect (Fig. 1, *A*–*C*). This indicates that in these oocytes, the channels present at the cell surface were fully cleaved. Thus, coexpression of ENaC and TMPRSS2 strongly activates ΔI_ami_ and mimics proteolytic ENaC activation by chymotrypsin in oocytes expressing ENaC alone.

It has been shown that mutating the catalytic triad of TMPRSS4 (CAP2) or matriptase (CAP3) abolished the stimulatory effect of these membrane-anchored proteases on ENaC (41, 45). Therefore, we used a similar strategy to investigate whether the catalytic activity of TMPRSS2 is required for its stimulatory effect on ENaC. In these experiments, ENaC was coexpressed with catalytically inactive TMPRSS2^S441A^, in which the critical serine residue belonging to the catalytic triad was substituted by an alanine. Importantly, TMPRSS2^S441A^ failed to stimulate ENaC (Fig. 1*A*, *right panel*; Fig. 1*B*). Moreover, the relative stimulatory effect of chymotrypsin in oocytes coexpressing ENaC and TMPRSS2^S441A^ was not significantly different from that observed in control oocytes expressing ENaC alone (Fig. 1*C*). Thus, the stimulatory effect of TMPRSS2 coexpression on ENaC-mediated currents depends on the proteolytic activity of TMPRSS2.

In parallel experiments, we assessed proteolytic activity at the cell surface of oocytes using an established fluorogenic substrate assay (56, 67). Previous substrate specificity analysis demonstrated that TMPRSS2 preferentially cleaves substrates with an arginine (R) residue at the P1 position (62). Therefore, we predicted that the fluorogenic substrate Boc–QAR–AMC (*t*-butyloxycarbonyl–Gln-Ala-Arg–7-amino-4-methylcoumarin; R&D Systems) (68), which we previously used to detect trypsin-like proteolytic activity at the cell surface of PRSS8 (prostasin; CAP1)–expressing oocytes (56), can also be used to detect TMPRSS2-dependent proteolytic activity. Indeed, as shown in Figure 1*D*, a strong increase of the fluorescence signal over time was observed in oocytes coexpressing ENaC and TMPRSS2. Thus, heterologous expression of human TMPRSS2 resulted in the appearance of strong proteolytic activity at the cell surface of oocytes. In contrast, this was not observed in oocytes coexpressing ENaC with TMPRSS2^S441A^ or in oocytes expressing ENaC alone. The latter finding confirms our previously reported observation (56) that endogenous trypsin-like protease activity is below the detection limit at the cell surface of control oocytes.

To exclude that the lack of proteolytic activity and ENaC activation was due to impaired protein expression or trafficking of TMPRSS2^S441A^, we analyzed intracellular and cell surface expression of wildtype TMPRSS2 and TMPRSS2^S441A^ using western blot analysis and a biotinylation approach (Fig. 1*E*). In the intracellular protein fraction (Fig. 1*E*, *left panels*), wildtype TMPRSS2 was detected predominantly as ∼27 kDa band most likely representing the catalytic chain of mature TMPRSS2 (69). In comparison, the intensity of the band corresponding to the zymogen form (∼65 kDa) of TMPRSS2 was much weaker. In contrast, we observed strong intracellular expression of the zymogen (∼65 kDa) form of TMPRSS2^S441A^, whereas the mature (∼27 kDa) form of TMPRSS2^S441A^ could hardly be detected. This latter finding is in good agreement with previous reports suggesting autocatalytic processing of TMPRSS2, which can be blocked by the S441A mutation (69). As expected, no TMPRSS2-specific bands were detected in oocytes expressing ENaC alone, neither intracellularly nor at the cell surface. At the cell surface of ENaC- and TMPRSS2-coexpressing cells, wildtype TMPRSS2 could be detected in its mature (*i.e.*, active) ∼27 kDa form (Fig. 1*E*, *right panels*) consistent with our protease activity measurements with Boc–QAR–AMC. Interestingly, in oocytes coexpressing ENaC and TMPRSS2^S441A^, the mutant protein was detectable at the cell surface in both mature (∼27 kDa) and zymogen (∼65 kDa) forms. Moreover, cell surface expression of the catalytically inactive TMPRSS2^S441A^ was overall stronger than that of wildtype TMPRSS2. We have no explanation for the increased cell surface expression of the mutant protein. Nevertheless, our findings demonstrate that the lack of ENaC stimulation by coexpression of TMPRSS2^S441A^ is not because of impaired protein expression or plasma membrane trafficking of the mutant protease. Thus, the failure of TMPRSS2^S441A^ to stimulate ENaC currents is most likely because of the absence of its proteolytic activity.

Note, that in the experiments shown in Figure 1, we used a TMPRSS2 construct with a C-terminal hemagglutinin (HA) tag (see Experimental procedures section) to allow reliable detection of TMPRSS2 by western blot analysis (Fig. 1*E*). Therefore, we performed control experiments coexpressing ENaC with untagged TMPRSS2. These experiments confirmed the stimulatory effect of coexpressed TMPRSS2 on ENaC and also the appearance of trypsin-like proteolytic activity at the cell surface of TMPRSS2-coexpressing oocytes (Fig. S1).

### The stimulatory effect of TMPRSS2 on ENaC is due to a large increase of channel open probability

**Figure 2:**
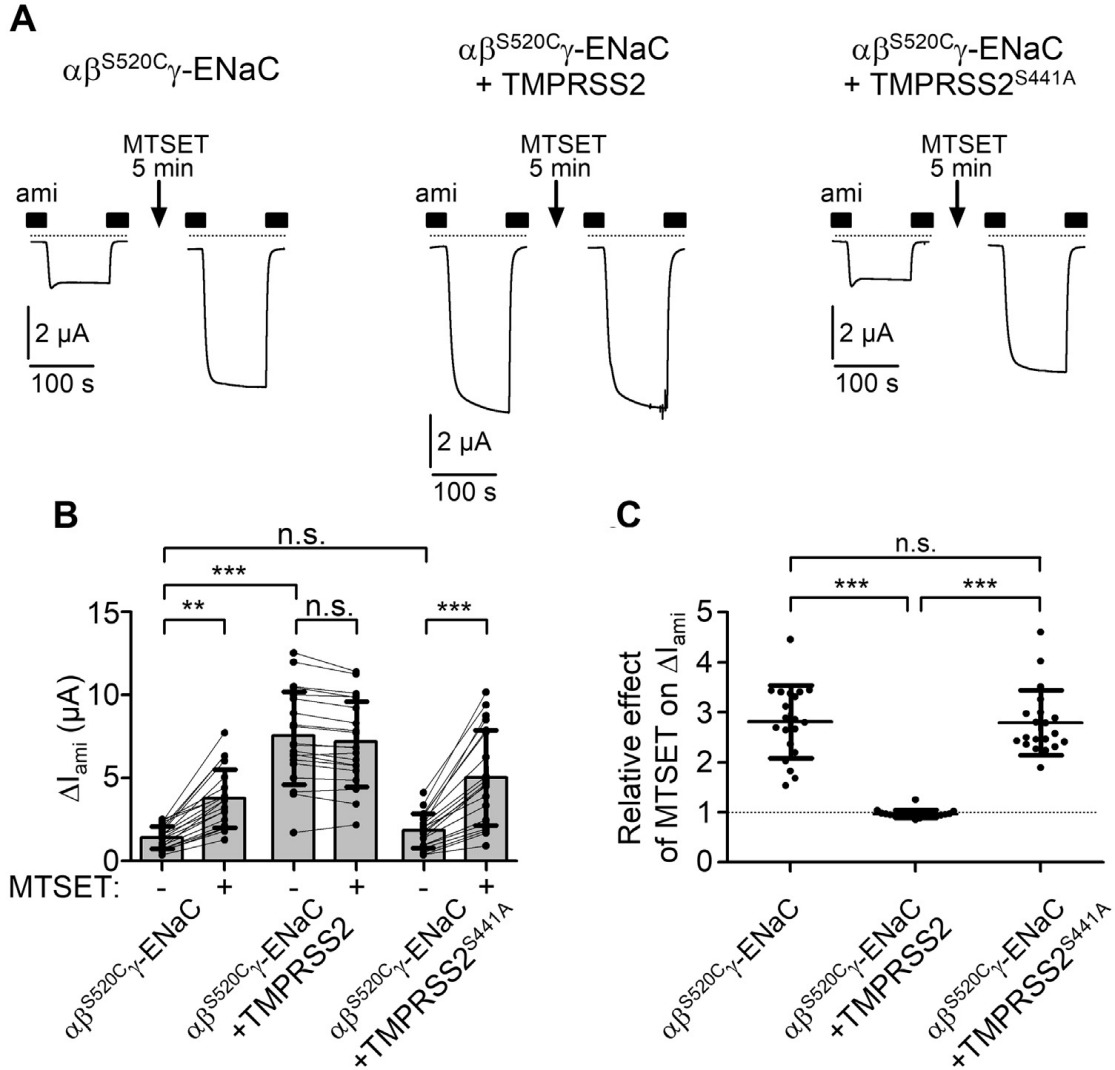
**Coexpression of ENaC with TMPRSS2 largely increases average channel open probability.***A*, representative whole-cell current traces are shown for oocytes expressing wildtype α-ENaC and γ-ENaC together with a mutant β-ENaC subunit carrying a single-point mutation (S520C) without TMPRSS2 (*left traces*, αβ^S520C^γ-ENaC), with coexpression of wildtype TMPRSS2 (*middle traces*, αβ^S520C^γ-ENaC + TMPRSS2) or catalytically inactive TMPRSS2 (*right traces*, αβ^S520C^γ-ENaC + TMPRSS2^S441A^). In each individual oocyte, current measurement was performed before and after 5 min of incubation in ND96 bath solution containing MTSET (1 mM) and amiloride (ami; 2 μM). For the current measurement, an oocyte was clamped at a holding potential of −60 mV. The oocyte was unclamped during the incubation time in the presence of MTSET to minimize sodium loading of the oocytes. Before the second current measurement, MTSET was washed out with ND96 containing 2 μM ami. Impaling microelectrodes were not removed from the oocyte until the end of the experiment. The presence of ami (2 μM) in the bath solution is indicated by *filled bars*. *Dashed lines* indicate zero current level. *B*, summary of ΔI_ami_ values obtained in similar experiments as shown in (*A*). *Lines* connect data points obtained in an individual oocyte. Mean ± SD and data points for individual oocytes are shown; ∗∗∗*p* < 0.001; ∗∗*p* < 0.01; ns, one-way ANOVA with Bonferroni post hoc test (n = 20, N = 3). *C*, relative stimulatory effect of MTSET on ΔI_ami_ summarized from data shown in (*B*). *Dashed line* indicates normalized ΔI_ami_ value of one (no effect). Mean ± SD and data points for individual oocytes are shown; ∗∗∗*p* < 0.001; ns, Kruskal–Wallis with Dunn’s post hoc test. ENaC, epithelial sodium channel; MTSET, 2-(trimethylammonium)ethyl methanethiosulfonate bromide; ns, not significant; TMPRSS2, transmembrane serine protease 2.

Proteolytic channel activation by TMPRSS2 is likely to cause an increase of ENaC open probability (*P*_O_). To investigate this, we used an established approach to assess average ENaC *P*_O_ in the oocyte expression system (9, 23, 70). For this purpose, wildtype α-ENaC and γ-ENaC subunits were coexpressed with a mutant β-ENaC subunit carrying a cysteine substitution at the so-called degenerin site (β^S520C^). This cysteine residue can be covalently modified by the sulfhydryl reagent 2-(trimethylammonium)ethyl methanethiosulfonate bromide (MTSET). This has been shown to increase the *P*_O_ of mutant αβ^S520C^γ-ENaC close to 1 (71, 72). As illustrated in Figure 2*A* (*left traces*) and summarized in Figure 2, *B* and *C*, application of MTSET for 5 min strongly stimulated ΔI_ami_ in oocytes expressing αβ^S520C^γ-ENaC by approximately threefold, which is consistent with our recently published data obtained using a similar experimental protocol (70). Assuming that MTSET increased *P*_O_ of all channels close to 1, average baseline *P*_O_ of αβ^S520C^γ-ENaC was ∼0.3. This is in good agreement with previously published baseline *P*_O_ values for human ENaC expressed in oocytes (70, 73). Importantly, no stimulatory effect of MTSET was observed in oocytes coexpressing αβ^S520C^γ-ENaC and TMPRSS2 (Fig. 2*A*, *middle traces*; Fig. 2, *B* and *C*). This indicates, that in oocytes coexpressing TMPRSS2, average ENaC *P*_O_ is nearly maximal and cannot be further increased by the application of MTSET. Moreover, the increased *P*_O_ of ENaC because of TMPRSS2 coexpression can fully explain the stimulatory effect of TMPRSS2 on ΔI_ami_. In contrast, in oocytes coexpressing αβ^S520C^γ-ENaC and TMPRSS2^S441A^, the stimulatory effect of MTSET was similar to that observed in control oocytes expressing αβ^S520C^γ-ENaC alone (Fig. 2*A*, *right traces*; Fig. 2, *B* and *C*). Thus, catalytically inactive TMPRSS2^S441A^ does not increase ENaC *P*_O._

Taken together, our findings indicate that the stimulatory effect of TMPRSS2 on ENaC is mainly because of a large increase of *P*_O_. Moreover, these experiments confirm that proteolytic activity of TMPRSS2 is essential for its stimulatory effect on ENaC, suggesting that TMPRSS2 cleaves ENaC directly.

### The stimulatory effect of TMPRSS2 on ENaC is partially preserved when proteolytic activity at the cell surface is inhibited by aprotinin

According to the current paradigm, membrane-anchored proteases perform the final activating cleavage in γ-ENaC when the channel reaches the plasma membrane (14). To test whether TMPRSS2-dependent proteolytic ENaC activation occurs at the cell surface, we preincubated oocytes for 48 h after complementary RNA (cRNA) injection with or without aprotinin (100 μg/ml). In experiments with aprotinin-pretreated oocytes, aprotinin was also present in the bath solution throughout the whole-cell current recordings. Aprotinin inhibits a wide range of trypsin-like serine proteases (43, 50, 51, 74, 75). Thus, it can be assumed that aprotinin also inhibits TMPRSS2. Aprotinin is a polypeptide consisting of 58 amino acid residues with a molecular mass of about 6.5 kDa. This makes it unlikely that it easily permeates the cell membrane. Thus, the inhibitory effect of aprotinin is probably limited to extracellular soluble and membrane-bound proteases. We cannot exclude the possibility that over time a portion of aprotinin may be internalized, for example, by endocytosis. However, after internalization, aprotinin is likely to be degraded *via* the proteasomal/lysosomal pathway. Therefore, aprotinin is likely to block the catalytic activity of TMPRSS2 mainly at the cell surface without appreciable effect on its intracellular activity.

**Figure 3:**
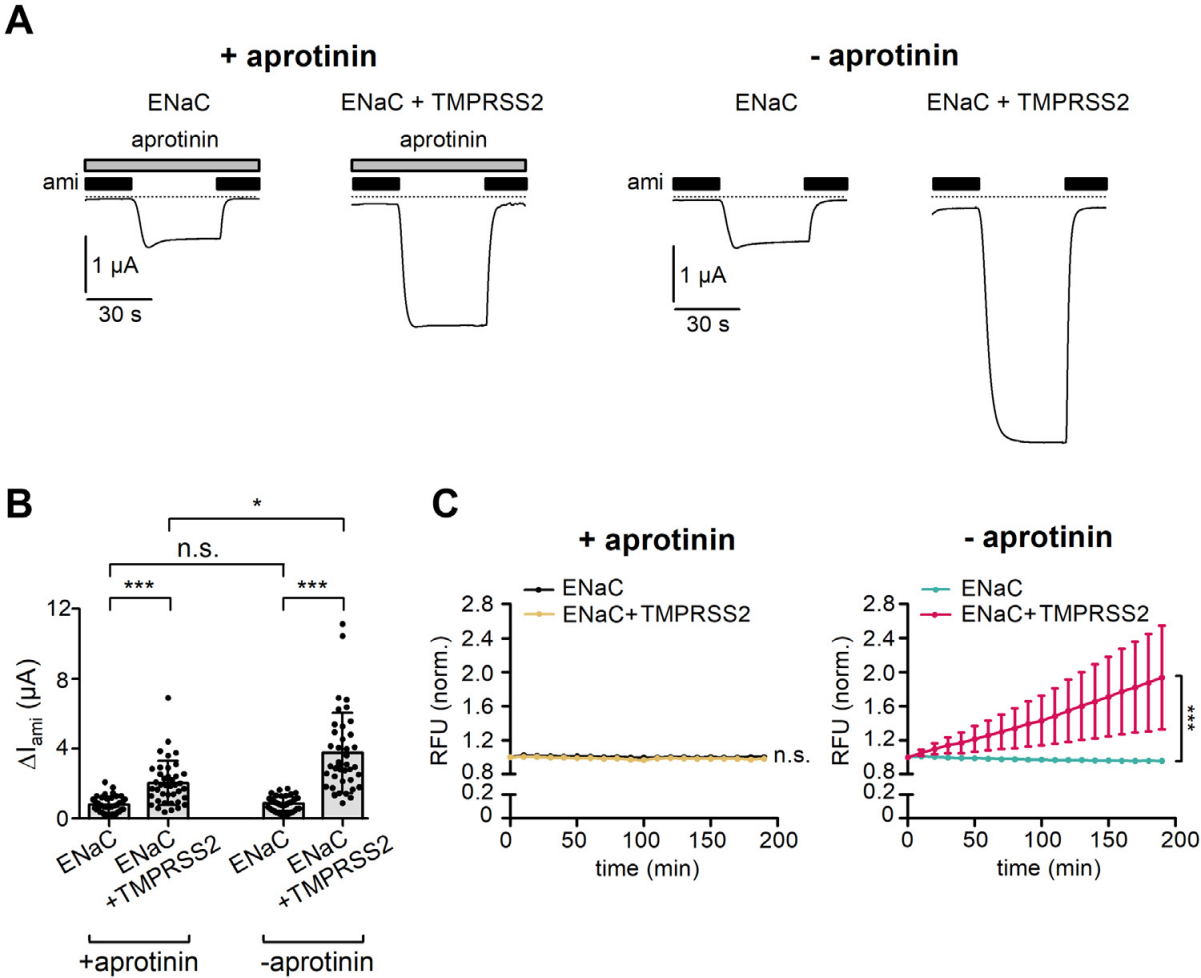
**Aprotinin abolishes the proteolytic activity of TMPRSS2 at the cell surface but not its stimulatory effect on ENaC.***A*, representative whole-cell current traces are shown for oocytes expressing ENaC without or with coexpression of TMPRSS2, as indicated. After cRNA injection, oocytes were incubated for 48 h in standard incubation solution with aprotonin (*two left panels*, + aprotinin, 100 μg/ml) or without aprotinin (*two right panels*, − aprotinin). In experiments with aprotinin-pretreated oocytes, aprotinin (100 μg/ml) was also present in the bath solution throughout the whole-cell current recordings as indicated by *gray bars*. Amiloride (ami, 2 μM) was present in the bath solution as indicated by *black bars*. *Dashed lines* indicate zero current level. *B*, summary of ΔI_ami_ values obtained in similar experiments as shown in (*A*). Mean ± SD and data points for individual oocytes are shown; ∗∗∗*p* < 0.001; ∗*p* < 0.05; ns, one-way ANOVA with Bonferroni post hoc test (n = 40, N = 3). *C*, progress curves of trypsin-like proteolytic activity at the cell surface (RFU = relative fluorescent unit; mean ± SD) were obtained as described for Figure 1*D* in parallel experiments with oocytes from the same batches as shown (*A* and *B*). ∗∗∗*p* < 0.001; ns, Kruskal–Wallis with Dunn’s post hoc test (at the time point 190 min, n = 19, N = 3). cRNA, complementary RNA; ENaC, epithelial sodium channel; ns, not significant; TMPRSS2, transmembrane serine protease 2.

Interestingly, a substantial stimulatory effect of TMPRSS2 coexpression on ΔI_ami_ was preserved in oocytes maintained in the continuous presence of aprotinin (Fig. 3, *A* and *B*). Indeed, in the presence of aprotinin, ΔI_ami_ averaged 2.0 ± 1.3 μA (n = 40) in TMPRSS2- and ENaC-coexpressing oocytes compared with 0.8 ± 0.5 μA (n = 40, *p* < 0.001) in oocytes expressing ENaC alone. In control experiments performed in parallel in the absence of aprotinin, the stimulatory effect of TMPRSS2 was even more pronounced with ΔI_ami_ averaging 3.7 ± 2.3 μA (n = 40) in oocytes coexpressing TMPRSS2 and 0.8 ± 0.4 μA (n = 40, *p* < 0.001) in oocytes expressing ENaC alone (Fig. 3, *A* and *B*). This suggests that in the presence of aprotinin, proteolytic ENaC activation by coexpressed TMPRSS2 is incomplete and that cell surface protease activity contributes, at least in part, to channel activation by TMPRSS2 in control cells maintained without aprotinin.

In oocytes expressing ENaC alone, incubation with aprotinin did not significantly alter baseline ΔI_ami_ (Fig. 3, *A* and *B*). This is consistent with the finding that no measurable aprotinin-sensitive trypsin-like proteolytic activity was observed at the cell surface of these oocytes (Fig. 3*C*). In contrast, the substantial trypsin-like proteolytic activity at the cell surface of oocytes coexpressing TMPRSS2 and ENaC was completely abolished by aprotinin (Fig. 3*C*). Thus, the partially preserved stimulatory effect of TMPRSS2 on ENaC in oocytes treated with aprotinin is unlikely to be due to residual proteolytic activity at the cell surface. Instead, it is probably because of intracellular proteolytic activity of TMPRSS2, which activates ENaC and is not affected by extracellular aprotinin.

### TMPRSS2 coexpression leads to the appearance of fully cleaved γ-ENaC in the intracellular and cell surface protein fraction

**Figure 4:**
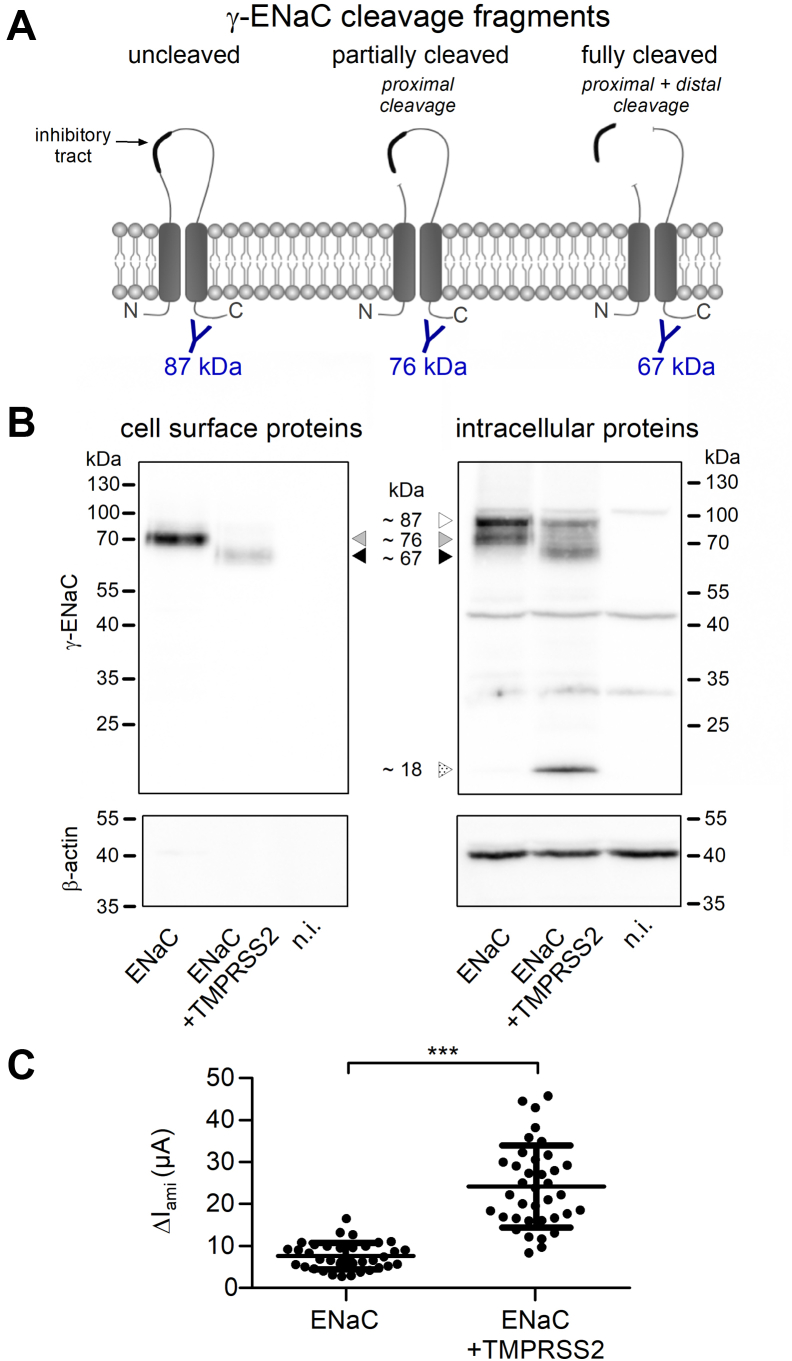
**TMPRSS2-dependent ENaC stimulation was associated with the appearance of fully cleaved γ-ENaC in the intracellular and cell surface protein fraction.***A*, schematic diagram showing γ-ENaC cleavage fragments, which can be detected using an antibody (in *blue*) raised against a C-terminal γ-ENaC epitope. The expected molecular weights of the corresponding C-terminal γ-ENaC cleavage fragments are given below. *B*, representative western blots showing cell surface (*left upper panel*) or intracellular (*right upper panel*) expression of γ-ENaC in oocytes from one batch expressing ENaC alone or coexpressing ENaC with TMPRSS2. Specific signal of γ-ENaC was detected using an antibody against the C-terminal epitope of γ-ENaC. To increase ENaC expression and improve γ-ENaC detection in western blot experiments, oocytes were injected with more than the usual amount of cRNA for ENaC (1 ng/subunit/oocyte) and TMPRSS2 (5 ng/oocyte). Noninjected oocytes served as a control (n.i.). Uncleaved (∼87 kDa), partially cleaved (∼76 kDa), and fully cleaved γ-ENaC (∼67 kDa) are indicated by *open*, *gray*, and *black-filled arrowheads*, respectively. A putative γ-ENaC degradation product (∼18 kDa) is indicated by an *open arrowhead* with *dot pattern*. To validate separation of cell surface proteins from intracellular proteins, blots were stripped and reprobed using an antibody against β-actin (*lower panels*). Similar results were obtained in four additional repeats (n = 5). *C*, in parallel experiments to those shown in (*B*), ΔI_ami_ values were measured to confirm the stimulatory effect of TMPRSS2 on ENaC in these batches of oocytes (n = 37, N = 5). Note that the relative stimulatory effect of TMPRSS2 on ΔI_ami_ was similar to that for Figure 1, Figure 2, Figure 3, but the absolute current values were higher, which reflects increased ENaC expression because of the larger amount of cRNA injected in these experiments. Mean ± SD and data points for individual oocytes are shown; ∗∗∗*p* < 0.001; two-tailed unpaired Student’s *t* test. cRNA, complementary RNA; ENaC, epithelial sodium channel; TMPRSS2, transmembrane serine protease 2.

To detect γ-ENaC and its typical cleavage products (Fig. 4*A*) in the intracellular protein fraction and at the cell surface of oocytes with and without coexpression of TMPRSS2, we used western blot analysis in combination with a biotinylation approach. In the nonbiotinylated intracellular protein fractions, a ∼87 kDa γ-ENaC band corresponding to uncleaved γ-ENaC could be detected in oocytes with and without TMPRSS2 coexpression (Fig. 4*B*, *right* *panel*). As expected from previous studies, an additional prominent ∼76 kDa band was present in the intracellular protein fraction from oocytes expressing ENaC alone. This cleavage product is thought to result from partial γ-ENaC cleavage by endogenous furin or furin-like convertases at the so-called furin cleavage site (R138) and can be detected using an antibody recognizing a C-terminal γ-ENaC epitope (Fig. 4*A*) (9, 21, 23, 76). Importantly, coexpression of TMPRSS2 largely reduced the intensity of the ∼76 kDa band in the intracellular protein fraction and resulted in the appearance of a ∼67 kDa band (Fig. 4*B*, *right* *panel*). This latter cleavage product has been shown to correspond to fully cleaved γ-ENaC associated with proteolytic channel activation (Fig. 4*A*) (22, 23, 24, 28). At the cell surface of ENaC- and TMPRSS2-coexpressing oocytes, we also detected mainly the ∼67 kDa band, whereas in oocytes expressing ENaC alone, the ∼76 kDa band was predominant (Fig. 4*B*, *left* *panel*). This is in good agreement with the robust stimulatory effect of TMPRSS2 coexpression on ΔI_ami_ observed in parallel electrophysiological experiments using oocytes from the same batches (Fig. 4*C*). TMPRSS2 coexpression resulted in the appearance of an additional small intracellular γ-ENaC band of ∼18 kDa (Fig. 4*B*, *right* *panel*), which was not detected at the cell surface (Fig. 4*B*, *left* *panel*), and may correspond to a degradation product. A possible functional role of this additional γ-ENaC cleavage product remains to be elucidated.

Taken together, the functional experiments with aprotinin and the western blot results support the hypothesis that TMPRSS2 cleaves γ-ENaC not only at the cell surface but at least in part also intracellularly before the channel reaches the plasma membrane.

### The stimulatory effect of TMPRSS2 on ENaC is due to proteolytic release of the γ-inhibitory tract

**Figure 5:**
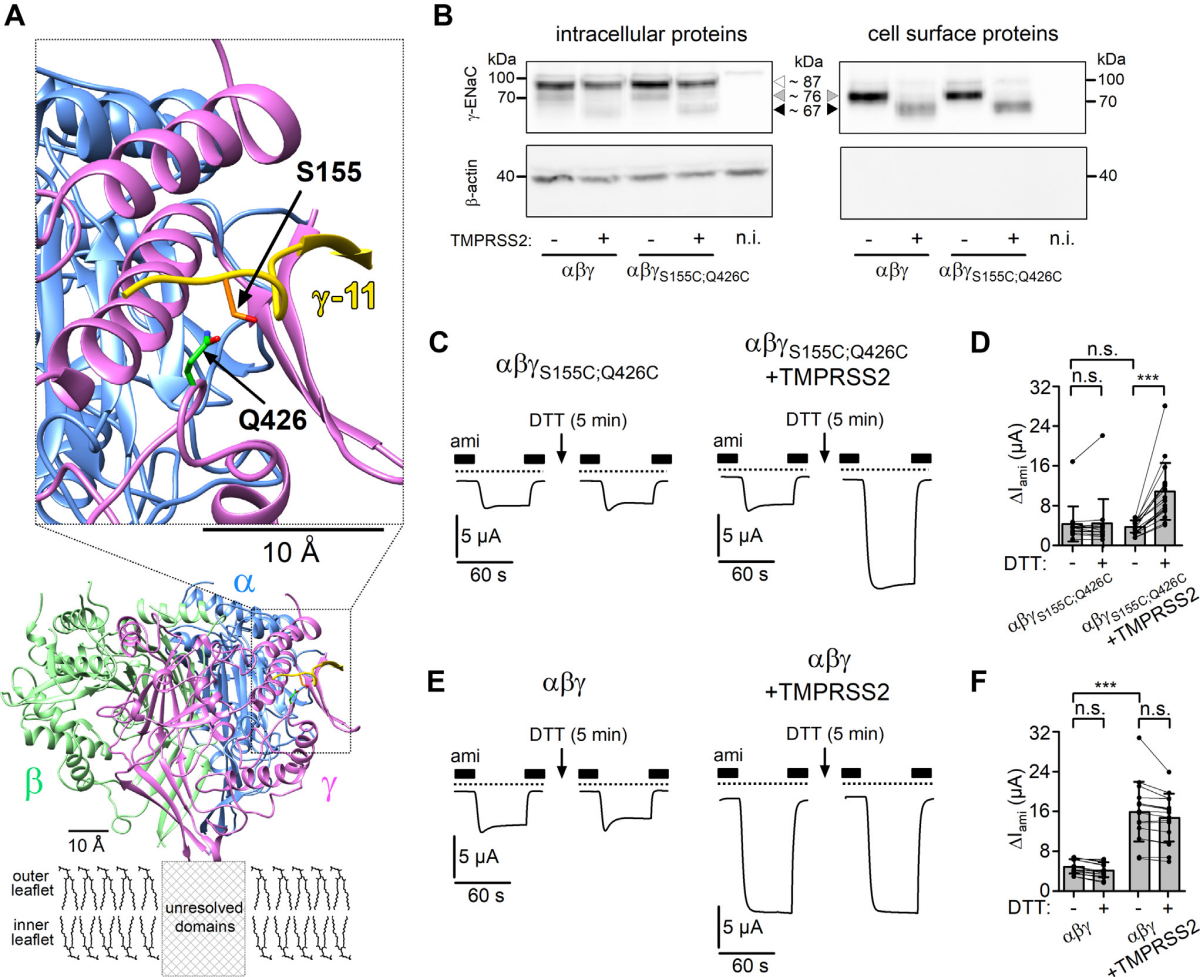
**Preventing the release of the inhibitory tract from γ-ENaC abolishes the stimulatory effect of TMPRSS2 on ENaC.***A*, *ribbon diagram* of extracellular domains of human αβγ-ENaC generated using atom coordinates from PDB entry 6WTH (8, 77). The putative location of unresolved transmembrane domains is indicated with a *box* placed within the plasma lipid bilayer (outer and inner leaflets), which is schematically depicted with dipalmitoylphosphatidylcholine (DPPC) molecules in *stick representation*. The *inset* shows the location of the specific binding site of the key inhibitory amino acid sequence (γ-11, in *yellow*) of the γ-inhibitory tract on an expanded scale. Serine (S155) and glutamine (Q426) residues, which were substituted by cysteines to introduce a disulfide bond between γ-inhibitory tract and its binding site, are indicated with *arrows* and shown in *stick representation* with side-chain carbons in *orange* for S155 or *green* for Q426, nitrogen in *blue*, and oxygens in *red*. Hydrogen atoms are omitted for clarity. *B*, representative western blots showing intracellular (*left upper panel*) or cell surface (*right upper panel*) expression of γ-ENaC in oocytes expressing wildtype (αβγ) or mutant (αβγ_S155C;Q426C_) ENaC without or with TMPRSS2 coexpression. Specific signal of γ-ENaC was detected using the same antibody as for Figure 4. Noninjected oocytes served as control (n.i.). Positions of uncleaved (∼87 kDa), partially cleaved (∼76 kDa), and fully cleaved γ-ENaC (∼67 kDa) are indicated by *open*, *gray*, and *black-filled arrowheads*, respectively. To validate separation of cell surface proteins from intracellular proteins, blots were stripped and reprobed using an antibody against β-actin (*lower panels*). Similar results were obtained in another repeat (n = 2). *C* and *E*, representative whole-cell current traces are shown for oocytes expressing the mutant ENaC (αβγ_S155C;Q426C_, *C*) or wildtype ENaC (αβγ, *E*) without (*left panels*) or with TMPRSS2 coexpression (*right panels*). In each individual oocyte currents were measured before and after 5 min incubation in ND96 bath solution containing DTT (30 mM) and amiloride (ami; 2 μM). The experimental protocol was similar to that described for Figure 2. The presence of ami (2 μM) is indicated by *filled bars*. *Dashed lines* indicate zero current level. *D* and *F*, summary of ΔI_ami_ values obtained in similar experiments as shown in (*C*) and (*E*). *Lines* connect data points obtained in an individual oocyte. Mean ± SD and data points for individual oocytes are shown; ∗∗∗*p* < 0.001; ns, Kruskal–Wallis with Dunn’s post hoc test (16≤ n ≤ 18, N = 3). ENaC, epithelial sodium channel; ns, not significant; PDB, Protein Data Bank; TMPRSS2, transmembrane serine protease 2.

The final cleavage of γ-ENaC in the region distal to the furin site causes the release of a γ-inhibitory tract consisting of ∼40 amino acid residues. The release of this γ-inhibitory tract is critical for channel activation (18). Extracellular application of a short synthetic peptide (γ-11), which corresponds to the 11 amino acid residues 153-RFSHRIPLLIF-163 located in the middle part of the γ-inhibitory tract, strongly inhibits ENaC-mediated currents probably by binding to a specific inhibitory binding site (17, 18). Indeed, recently published cryo-EM structures of the extracellular domains of human ENaC (7, 8, 77) revealed a specific binding site for this key inhibitory amino acid sequence. The binding site is formed by parts of the finger, thumb, and GRIP domains of γ-ENaC (Fig. 5*A*). The molecular structure of the binding site occupied by γ-11 suggests that the serine residue (S155) of γ-11 and the glutamine residue (Q426) of the binding site lie in close proximity to each other (Fig. 5*A*). We reasoned that substitution of these amino acid residues by cysteines (γ_S155C;Q426C_) would lead to the formation of an additional disulfide bond between the γ-inhibitory tract and its binding site. Moreover, we hypothesized that stabilizing the interaction of the γ-inhibitory tract to its binding site by this additional disulfide bond would prevent channel activation despite proteolytic cleavage of γ-ENaC by TMPRSS2.

Western blot experiments performed under reducing conditions (see the Experimental procedures section) confirmed that overall intracellular and cell surface expression of mutant γ_S155C;Q426C_-ENaC was similar to that of wildtype γ-ENaC with or without TMPRSS2 coexpression (Fig. 5*B*). Moreover, cleavage of the mutant γ-subunit by TMPRSS2 coexpression was preserved intracellularly and at the cell surface (Fig. 5*B*). However, TMPRSS2 coexpression did not increase baseline ΔI_ami_ in oocytes with αβγ_S155C;Q426C_-ENaC (Fig. 5, *C* and *D*) unlike in matched oocytes with wildtype αβγ-ENaC, where TMPRSS2 coexpression had its usual stimulatory effect (Fig. 5, *E* and *F*). This suggests that despite γ-ENaC cleavage, the γ-inhibitory tract remains attached to its binding site because of the introduced disulfide bond, thereby preventing activation of the mutant channel. Disulfide bonds can be reduced by incubating oocytes with agents like DTT (78). Indeed, incubation of oocytes coexpressing αβγ_S155C;Q426C_-ENaC and TMPRSS2 with DTT (30 mM, 5 min) increased ΔI_ami_ by approximately threefold (Fig. 5, *C* and *D*). This stimulatory effect was similar to that of TMPRSS2 on baseline ΔI_ami_ in wildtype ENaC (Fig. 5, *E* and *F*). DTT had no stimulatory effect on ΔI_ami_ in oocytes expressing αβγ_S155C;Q426C_-ENaC alone or in oocytes expressing wildtype αβγ-ENaC with or without TMPRSS2 coexpression (Fig. 5, *C*–*F*). This indicates that the observed DTT-mediated stimulation of ΔI_ami_ in oocytes coexpressing αβγ_S155C;Q426C_-ENaC and TMPRSS2 was due to the specific reduction of the disulfide bond between S155C and Q426C in γ-ENaC and the release of the γ-inhibitory tract from γ-ENaC, which had been cleaved by TMPRSS2 as evidenced by our western blot data (Fig. 5*B*).

To conclude, these data clearly demonstrate that the stimulatory effect of TMPRSS2 on ENaC is due to proteolytic cleavage of γ-ENaC and subsequent release of the γ-inhibitory tract.

### Docking simulations suggest that TMPRSS2 cleaves γ-ENaC at multiple sites distal to the γ-inhibitory tract

**Figure 6:**
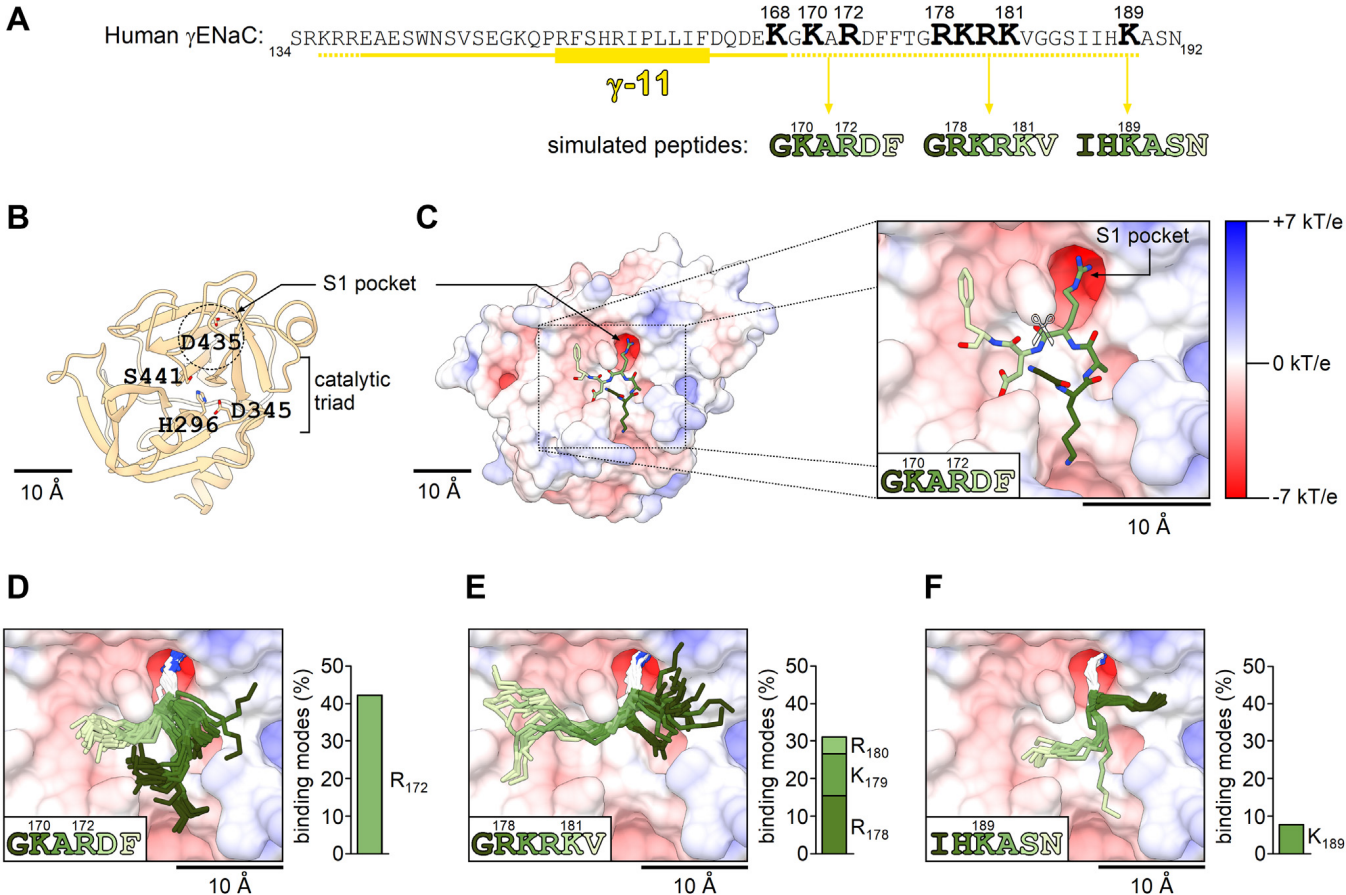
**Prediction of putative TMPRSS2 cleavage sites distal to the γ-inhibitory tract using a molecular docking approach.***A*, the primary sequence of human γ-ENaC (amino acid residues 134–192) is shown in the region including the γ-inhibitory tract (*underlined in yellow*). The key inhibitory amino acid sequence (γ-11) is highlighted with a *yellow rectangle*. Candidate TMPRSS2 cleavage sites (arginine and lysine residues) distal to the γ-inhibitory tract are in *bold* and *numbered*. The length of the amino acid sequence released by cleavage may slightly vary depending on the cleavage site used in the region indicated by the *dashed yellow line*. Sequences of three 6-mer peptides, which were docked to the catalytic domain of TMPRSS2 in computer simulations, are shown below the γ-ENaC sequence. The numbering of amino acid residues in the simulated peptides is the same as in the γ-ENaC sequence. *B* and *C*, a homology model of human TMPRSS2 generated based on the crystal structure of human homologous protease hepsin (PDB accession no.: 1Z8G) is shown in *ribbon* (*B*) or electrostatic potential molecular surface representation (*C*). In (*B*), amino acid residues forming the catalytic triad (histidine H296, aspartate D345, and serine S441) and the aspartate residue D435 at the bottom of the S1 pocket are shown in *stick representation* with carbons in *tan*, nitrogens in *blue*, and oxygens in *red*. In (*C*), a representative binding mode of the GKARDF-peptide to the TMPRSS2 catalytic domain (in the *inset* on an expanded scale), which fulfills the selection criteria described in Figure S2*A*, is depicted in *stick representation* with carbons in the same color as the corresponding amino acid residue of the peptide sequence given in the *lower left corner* of the *inset*, nitrogens in *blue*, and oxygens in *red*. Hydrogen atoms are omitted for clarity. Scissile peptide bond is marked in the *inset* with a *scissors symbol* (✄). *D*–*F*, all binding modes that fulfill the selection criteria are shown for the GKARDF-peptide (*D*), GRKRKV-peptide (*E*), or IHKASN-peptide (*F*). Peptide backbone carbons and nitrogens (in the same color as the corresponding amino acid residue of the peptide sequence given in the *lower left corner*) and the side chains of arginine or lysine residues occupying the S1 pocket (with carbons in *white* and nitrogens in *blue*) are shown. *Bar diagrams* demonstrate the percentage of binding modes, which fulfill the selection criteria out of the total number of binding modes (90) generated for each peptide, and indicate the arginine or lysine residue that occupies the S1 pocket. ENaC, epithelial sodium channel; PDB, Protein Data Bank; TMPRSS2, transmembrane serine protease 2.

Analysis of the γ-ENaC sequence revealed the presence of several positively charged residues, arginines and lysines, distal to the γ-inhibitory tract (Fig.6*A*), which may potentially play a role as TMPRSS2 cleavage sites. To assess whether TMPRSS2 has a preference for one of these putative cleavage sites, we used a molecular docking approach with three different 6-mer peptides corresponding to three segments of the γ-ENaC sequence: 169-GKARDF-174, 177-GRKRKV-182, and 187-IHKASN-192 (Fig. 6*A*). We simulated the binding of these 6-mer peptides to a homology model of the catalytic domain of human TMPRSS2 (Fig. 6, *B* and *C*). Importantly, the homology model of TMPRSS2 revealed a well-defined S1 pocket in the vicinity of the catalytic triad, which is highly negatively charged because of the presence of an aspartate residue (D435) at its bottom (Fig. 6, *B* and *C*). This corresponds well to the expected selectivity of TMPRSS2 for substrates with a positively charged arginine or lysine residue in the P1 position (62). To evaluate the results of the molecular docking simulations and to select binding modes that are likely to favor proteolysis from those that are incompatible with proteolysis, we defined two threshold selection criteria (Fig. S2*A*). First, the side chain of an arginine residue or a lysine residue in the P1 position of a peptide should occupy the S1 pocket and have a distance to the side chain of the aspartate (D435) at the bottom of the S1 pocket of less than 4 Å, compatible with the formation of a salt bridge between these two residues. Second, the distance between the carbonyl carbon atom of the scissile peptide bond and the hydroxyl group of the catalytic serine residue (S441) should be less than 4 Å, which is required for initiation of proteolysis (79). One representative binding mode of the GKARDF-peptide to the TMPRSS2 catalytic domain, which meets the aforementioned criteria, is shown in Figure 6*C*. Importantly, for the GKARDF-peptide, more than 40% of the generated binding modes fulfilled the selection criteria (Figs. 6*D* and S2*A*). In contrast to discarded binding modes, the selected binding modes were structurally similar and formed a prominent cluster (Fig. S2*A*). This was confirmed by calculation of root-mean-square deviation (RMSD) values between corresponding binding modes, which were on average significantly lower for binding modes that fulfilled the selection criteria compared with those calculated for binding modes that did not meet the selection criteria (Fig. S2*E*). This indicates that the strong P1–S1 interaction stabilizes the backbone conformation of the whole GKARDF-peptide within the catalytic domain of TMPRSS2. Interestingly, we observed that only the side chain of the arginine but not that of the lysine occupied the S1 pocket of TMPRSS2, which is consistent with the preference of TMPRSS2 for arginine over lysine observed experimentally (62). To summarize, computer modeling suggests that TMPRSS2 may cleave γ-ENaC in the 169-GKARDF-174 region, most probably after the arginine residue (R172). Next, we simulated the interaction of the GRKRKV-peptide with TMPRSS2 (Fig. 6*E*). Overall, more than 30% of the generated binding modes fulfilled the selection criteria, suggesting that TMPRSS2 may effectively cleave γ-ENaC in the 177-GRKRKV-182 region, most likely after the arginine residue R178, but possibly also after K179 or R180. Finally, we simulated the binding of the IHKASN-peptide to the catalytic domain of TMPRSS2. As demonstrated in Figure 6*F*, the lysine side chain occupied the S1 pocket of TMPRSS2, but this was observed only in less than 10% of all generated binding modes. This finding suggests that TMPRSS2 may cleave γ-ENaC also after the lysine residue (K189), but this cleavage site seems to be less preferable than R172 or R178.

In addition, we simulated how a substitution of a positively charged arginine or lysine residue within a putative cleavage site by an alanine may affect the interaction of γ-ENaC with TMPRSS2. This analysis was performed using the GKARDF-peptide. As shown in Fig. S2*B*, the substitution of the lysine residue by an alanine did not substantially affect the binding of the simulated peptide to the catalytic domain of TMPRSS2. Similar to the GKARDF-peptide, the mutant GAARDF-peptide also formed the close arginine–aspartate interaction within the S1 pocket in more than 40% of the generated binding modes (Fig. S2, *A* and *B*). In contrast, when the critical arginine residue was replaced by an alanine, as in the GKAADF-peptide, the lysine–aspartate interaction within the S1 pocket was observed only in less than 10% of all generated binding modes (Fig. S2*C*). Finally, the binding modes of the GAAADF-peptide lacking both positively charged residues demonstrated highly variable conformations (Fig. S2*D*) and high RMSD values (Fig. S2*E*) and generally resembled the discarded binding modes of the GKARDF-peptide (Fig. S2*A*), which would not favor proteolysis. In summary, these computer simulations predict that in the absence of the critical arginine (R172), TMPRSS2 may cleave γ-ENaC after an otherwise less preferable lysine residue (K170). Thus, TMPRSS2 may cleave γ-ENaC at multiple sites distal to the γ-inhibitory tract. Therefore, it may be necessary to replace several arginine and lysine residues in this region with alanines to fully abolish the stimulatory effect of TMPRSS2 on ENaC.

### TMPRSS2 cleaves γ-ENaC at multiple sites distal to the γ-inhibitory tract

**Figure 7:**
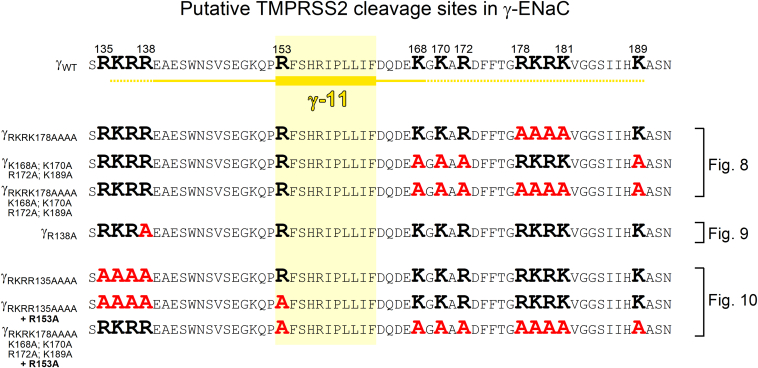
**γ-ENaC constructs generated by site-directed mutagenesis to identify TMPRSS2 cleavage sites distal and proximal to the γ-inhibitory tract.** Comparison of the primary sequence of wildtype γ-ENaC with those of γ-ENaC mutants generated and experimentally tested in the current study. The γ-inhibitory tract is labeled as described in Figure 6*A*. The putative TMPRSS2 cleavage sites (arginine and lysine residues) are marked in *bold black*. Putative cleavage sites eliminated by alanine substitutions are marked in *bold red*. Results from corresponding functional experiments are shown in Figure 8, Figure 9, Figure 10. ENaC, epithelial sodium channel; TMPRSS2, transmembrane serine protease 2.

**Figure 8:**
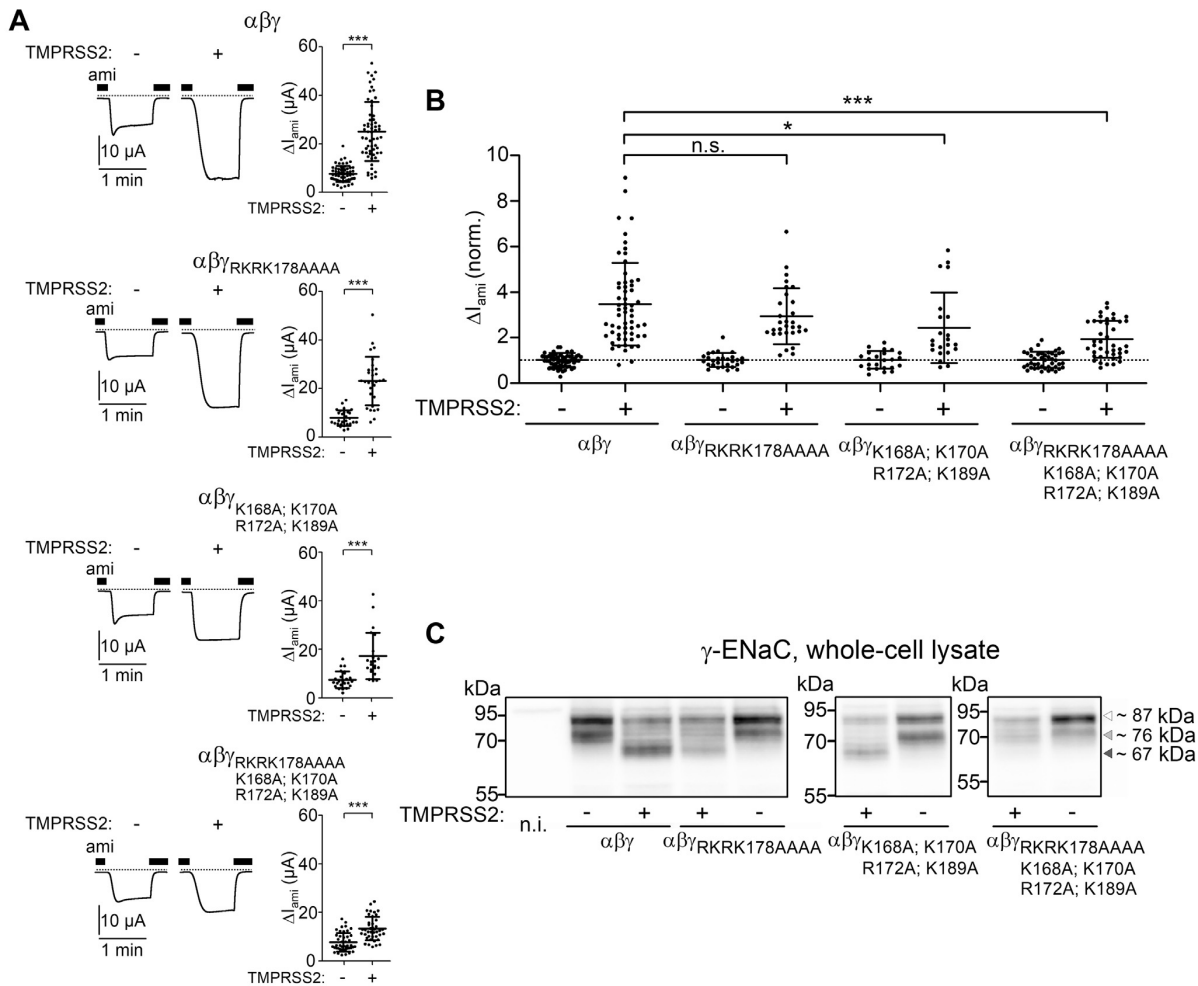
**Eliminating putative TMPRSS2 cleavage sites distal to the γ-inhibitory tract significantly reduces the stimulatory effect of TMPRSS2 coexpression on ENaC and prevents the appearance of fully cleaved γ-ENaC in whole-cell lysates.***A*, representative whole-cell current traces recorded in individual oocytes from the same batch injected with 1 ng/subunit/oocyte of cRNA for wildtype (αβγ) or mutant ENaC (αβγ_RKRK178AAAA_, αβγ_K168A;K170A;R172A;K189A_ or αβγ_RKRK178AAAA;K168A;K170A;R172A;K189A_) either alone (−) or in combination with 5 ng/oocyte cRNA for TMPRSS2 (+). Amiloride (ami, 2 μM) was present in the bath solution as indicated by *black bars*. *Dashed lines* indicate zero current level. Summary data obtained in similar experiments are shown to the *right* of the representative traces. Mean ± SD and data points for individual oocytes are shown; ∗∗∗*p* < 0.001; two-tailed unpaired Student’s *t* test (22≤ n ≤ 61, 3 ≤ N ≤ 9). *B*, ΔI_ami_ values of individual oocytes obtained in the same experiments as shown in (*A*) were normalized to the corresponding mean ΔI_ami_ recorded in oocytes from the same batch expressing wildtype (αβγ) or mutant ENaC (αβγ_RKRK178AAAA_, αβγ_K168A;K170A;R172A;K189A_, or αβγ_RKRK178AAAA;K168A;K170A;R172A;K189A_) without TMPRSS2 coexpression. *Dashed line* indicates a normalized ΔI_ami_ value of one (no effect). Mean ± SD and data points for individual oocytes are shown; ∗∗∗*p* < 0.001; ∗*p* < 0.05; ns; Kruskal–Wallis with Dunn’s post hoc test. *C*, representative western blots showing whole-cell expression of γ-ENaC detected using the same antibody as for Figure 4 in oocytes expressing wildtype (αβγ) or mutant ENaC (αβγ_RKRK178AAAA_, αβγ_K168A;K170A;R172A;K189A_, or αβγ_RKRK178AAAA;K168A;K170A;R172A;K189A_) either without (−) or with (+) TMPRSS2 coexpression. Noninjected oocytes served as control (n.i.). Uncleaved (∼87 kDa), partially cleaved (∼76 kDa), and fully cleaved γ-ENaC (∼67 kDa) are indicated by *open*, *light gray*, and *dark gray*–*filled arrowheads*, respectively. Similar results were obtained in additional repeats shown in Fig. S3. cRNA, complementary RNA; ENaC, epithelial sodium channel; ns, not significant; TMPRSS2, transmembrane serine protease 2.

To confirm our computer simulation results experimentally, we generated several mutant γ-ENaC subunits in which positively charged residues distal to the γ-inhibitory tract were substituted by alanines (Fig. 7). First, we mutated the putative prostasin cleavage site (γ_RKRK178AAAA_). Previously, it has been shown that mutating this site fully abolished the stimulatory effect of PRSS8 (prostasin) on ENaC (22). Interestingly, in αβγ_RKRK178AAAA_-ENaC–expressing oocytes, the stimulatory effect of TMPRSS2 on ΔI_ami_ was fully preserved and not significantly different from that in oocytes expressing wildtype αβγ-ENaC (Fig. 8*A*, *first and second panels from top*; and 8*B*). Thus, an intact prostasin site is not required for TMPRSS2-dependent ENaC activation. To test a possible involvement of alternative cleavage sites in this region, we generated another mutant (γ_K168A;K170A;R172A;K189A_) in which the prostasin site remained intact but three positively charged residues proximal to the prostasin site (K168, K170, and R172) and one lysine residue distal to the prostasin site (K189) were replaced with alanines. The stimulatory effect of TMPRSS2 on this mutant ENaC was only slightly reduced compared with that on wildtype ENaC (Fig. 8*A*, *third panel from top*; 8*B*). This indicates that TMPRSS2 can cleave γ-ENaC at the prostasin site when other positively charged residues distal to the γ-inhibitory tract are not available because of their replacement by alanines. Interestingly, when all arginine and lysine residues in the prostasin site and its vicinity were replaced with alanine residues (γ_RKRK178AAAA;K168A;K170A;R172A;K189A_), the stimulatory effect of TMPRSS2 on ENaC was largely abolished, albeit not completely (Fig. 8*A*, *lower panel*; 8*B*).

In parallel experiments, we analyzed the cleavage fragments of γ-ENaC in whole-cell lysates of oocytes expressing wildtype γ-ENaC or different γ-ENaC mutants. As illustrated by representative western blots shown in Figure 8*C* and similar western blots shown in Fig. S3, coexpression of TMPRSS2 resulted in a significant increase of the fully cleaved γ-ENaC band (∼67 kDa) in oocytes expressing wildtype γ-ENaC or the mutant subunits γ_RKRK178AAAA_ or γ_K168A; K170A; R172A; K189A_ but not in oocytes expressing γ_RKRK178AAAA; K168A; K170A; R172A; K189A_. This indicates that TMPRSS2 can efficiently cleave γ-ENaC distal to the γ-inhibitory tract, unless all positively charged residues are mutated in this region. The failure of TMPRSS2 to produce a detectable increase of the ∼67 kDa cleavage product in oocytes expressing γ_RKRK178AAAA; K168A; K170A; R172A; K189A_-ENaC is in good agreement with the largely reduced stimulatory effect of TMPRSS2 on ΔI_ami_ in this group of oocytes.

**Figure 10:**
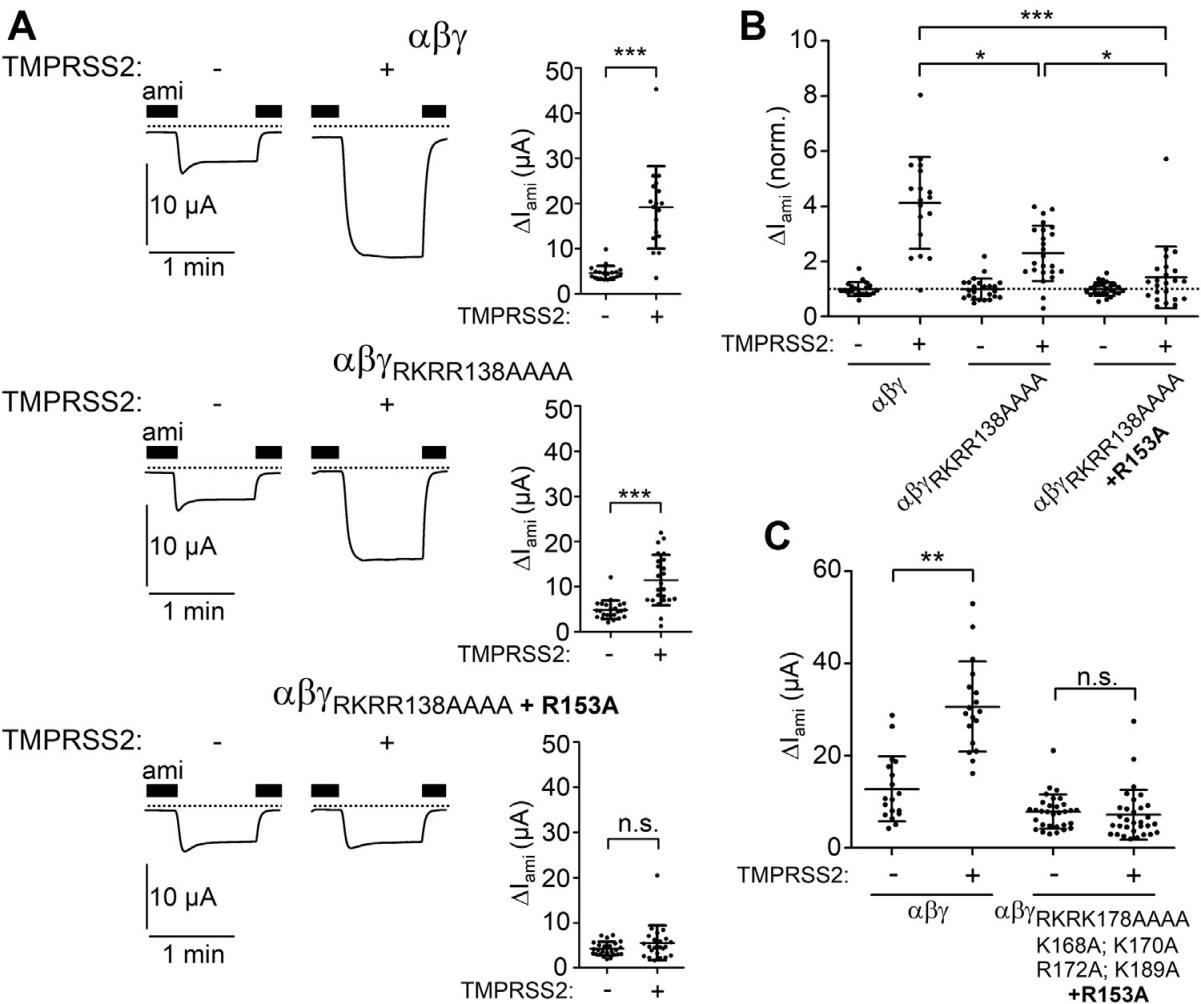
**The arginine residue R153 at the proximal end of γ-11 may serve as another TMPRSS2-cleavage site mediating partial ENaC activation.***A*, representative whole-cell current traces recorded in individual oocytes from the same batch injected with 1 ng/subunit/oocyte of cRNA for wildtype (αβγ) or mutant ENaC (αβγ_RKRR138AAAA_ or αβγ_RKRR138AAAA + R153A_) either alone (−) or in combination with 5 ng/oocyte cRNA for TMPRSS2 (+). Amiloride (ami, 2 μM) was present in the bath solution as indicated by *black bars*. *Dashed lines* indicate zero current level. Summary data obtained in similar experiments are shown to the *right* of the representative traces. Mean ± SD and data points for individual oocytes are shown; ∗∗∗*p* < 0.001; ns, two-tailed Mann–Whitney test (18 ≤ n ≤ 25, N = 3). *B*, ΔI_ami_ values of individual oocytes obtained in the same experiments as shown in (*A*) were normalized as described for Figure 8*B*. *Dashed line* indicates a normalized ΔI_ami_ value of one (no effect). Mean ± SD and data points for individual oocytes are shown; ∗∗∗*p* < 0.001; ∗*p* < 0.05; Kruskal–Wallis with Dunn’s post hoc test. *C*, summary of ΔI_ami_ values obtained in oocytes injected with 1 ng/subunit/oocyte of cRNA for wildtype (αβγ) or mutant ENaC (αβγ_RKRK178AAAA;K168A;K170A;R172A;K189A + R153A_) either alone (−) or in combination with 5 ng/oocyte cRNA for TMPRSS2 (+). Mean ± SD and data points for individual oocytes are shown; ∗∗*p* < 0.01; ns, Kruskal–Wallis with Dunn’s post hoc test. cRNA, complementary RNA; ENaC, epithelial sodium channel; TMPRSS2, transmembrane serine protease 2.

Collectively, our functional data confirm the computer modeling results and indicate that TMPRSS2 can cleave γ-ENaC at multiple sites distal to the γ-inhibitory tract without a clear preference for a particular position. Moreover, the preserved minor stimulatory effect of TMPRSS2 on αβγ_RKRK178AAAA; K168A; K170A; R172A; K189A_-ENaC suggests that additional TMPRSS2 cleavage sites exist in the γ-subunit, which may contribute to proteolytic channel activation (see later, Fig. 10*C*).

### TMPRSS2 can rescue γ-ENaC cleavage when the putative furin cleavage site R138 proximal to the γ-inhibitory tract is mutated

**Figure 9:**
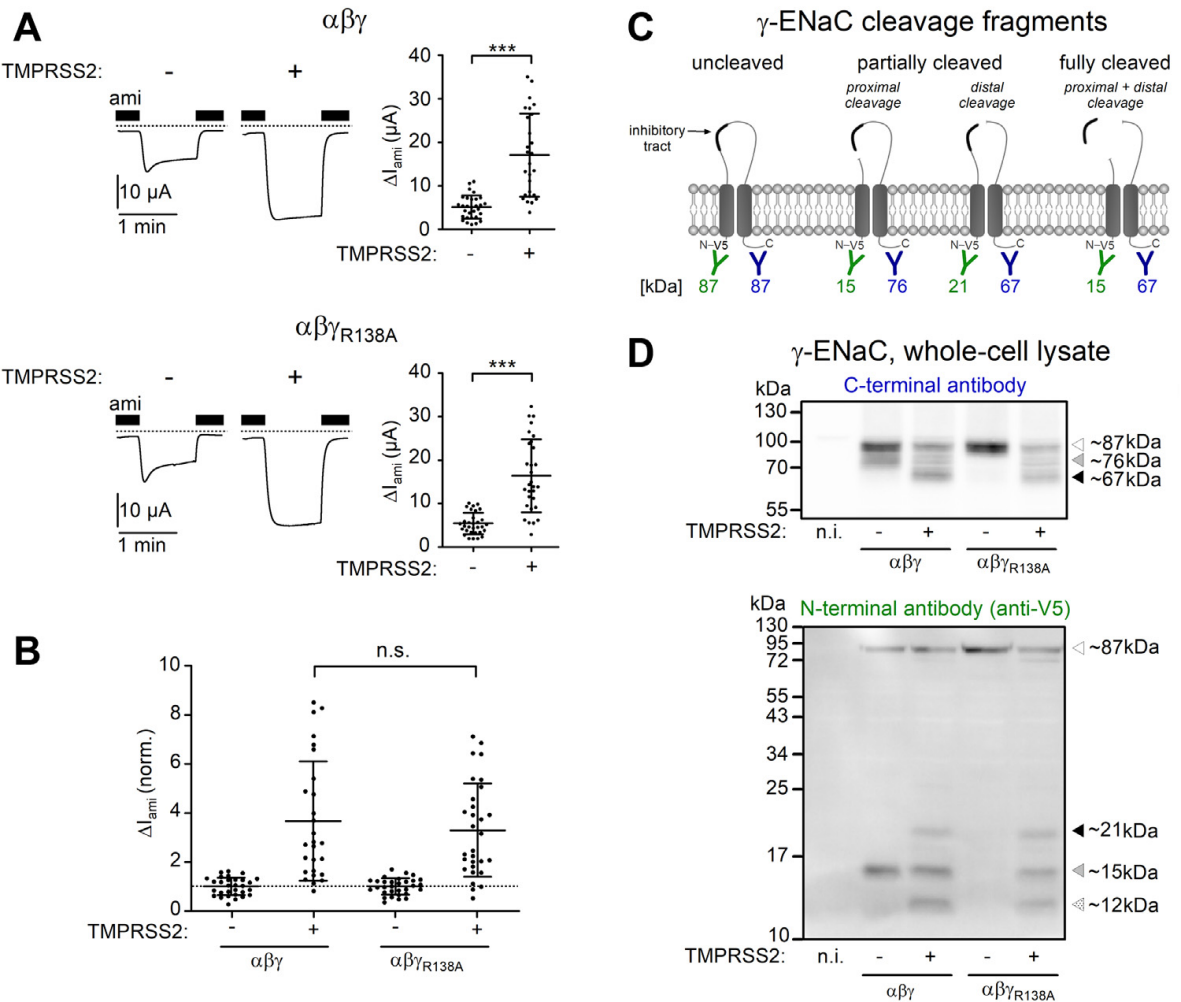
**TMPRSS2 cleaves γ-ENaC at sites proximal to the γ-inhibitory tract.***A*, representative whole-cell current traces recorded in individual oocytes from the same batch injected with 1 ng/subunit/oocyte of cRNA for wildtype (αβγ) or mutant ENaC (αβγ_R138A_) either alone (−) or in combination with 5 ng/oocyte cRNA for TMPRSS2 (+). V5-tag epitope was attached to the N terminus of γ-ENaC. Amiloride (ami, 2 μM) was present in the bath solution as indicated by *black bars*. *Dashed lines* indicate zero current level. Summary data obtained in similar experiments are shown to the *right* of the representative traces. Mean ± SD and data points for individual oocytes are shown; ∗∗∗*p* < 0.001; two-tailed unpaired Student’s *t* test (27≤ n ≤ 31, N = 5). *B*, ΔI_ami_ values of individual oocytes obtained in the same experiments as shown in (*A*) were normalized as described for Figure 8*B*. *Dashed line* indicates a normalized ΔI_ami_ value of one (no effect). Mean ± SD and data points for individual oocytes are shown; ns; one-way ANOVA with Bonferroni post hoc test. *C*, schematic diagram showing γ-ENaC cleavage fragments, which can be detected using an antibody raised against a C-terminal γ-ENaC epitope (in *blue*) or an anti-V5 antibody (in *green*). The expected molecular weights of the corresponding C-terminal and N-terminal γ-ENaC cleavage fragments are given below in the respective color. *D*, representative western blots showing whole-cell expression of γ-ENaC detected using the C-terminal anti-γ-ENaC (*upper panel*) or N-terminal anti-V5 antibody (*lower panel*) in oocytes expressing wildtype (αβγ) or mutant ENaC (αβγ_R138A_) either without (−) or with (+) TMPRSS2 coexpression. Noninjected oocytes served as control (n.i.). Uncleaved γ-ENaC (∼87 kDa), cleaved in the proximal (∼76 kDa and ∼15 kDa) or distal (∼67 kDa and ∼21 kDa) regions of the γ-inhibitory tract, are indicated by *open*, *gray*, and *black-filled arrowheads*, respectively. A putative N-terminal γ-ENaC degradation product (∼12 kDa) is indicated by an *open arrowhead* with *dot pattern*. Similar results were obtained in three additional repeats (n = 4). cRNA, complementary RNA; ENaC, epithelial sodium channel; ns, not significant; TMPRSS2, transmembrane serine protease 2.

The lack of a strong substrate specificity of TMPRSS2 (except that a positively charged arginine or lysine residue must be present at the P1 position) suggests that TMPRSS2 may cleave γ-ENaC also proximal to the γ-inhibitory tract in a polybasic region (135-RKRR-138; Fig. 7), which includes the putative furin cleavage site R138 (21, 23, 24, 76). As shown previously (Figs. 4, 5, and 8), cleavage at this site does not require coexpression of TMPRSS2 and is likely to be mediated by endogenous furin or furin-like convertases present in the oocyte expression system. Importantly, it can be prevented by replacing the arginine residue R138 with an alanine (γ_R138A_) (21, 22, 23, 76). We hypothesized that TMPRSS2 may be able to cleave αβγ_R138A_-ENaC after one of the remaining positively charged residues (R135, K136, and/or R137), thereby releasing the γ-inhibitory tract resulting in complete proteolytic ENaC activation. Indeed, in αβγ_R138A_-ENaC–expressing oocytes, the stimulatory effect of TMPRSS2 on ΔI_ami_ was similar to that in matched oocytes expressing wildtype αβγ-ENaC (Fig. 9, *A* and *B*). This indicates that TMPRSS2 can cleave mutant γ_R138A_-ENaC both proximally and distally to the γ-inhibitory tract. This conclusion was further supported by western blot analysis in which we used an antibody against a C-terminal γ-ENaC epitope (Fig. 9, *C* and *D*, *upper panel*) and in addition another antibody against a V5 tag attached to the N terminus of γ-ENaC (Fig. 9, *C* and *D*, *lower panel*). As expected, cleavage of γ-ENaC by endogenous proteases was effectively prevented by the R138A mutation. Indeed, in oocytes expressing αβγ_R138A_-ENaC, we detected neither a ∼76 kDa C-terminal fragment (Fig. 9*D*, *upper panel*) nor a corresponding ∼15 kDa N-terminal fragment (Fig. 9*D*, *lower panel*). In contrast, these fragments were present in matched αβγ-ENaC–expressing oocytes. Importantly, coexpression of TMPRSS2 and αβγ_R138A_-ENaC resulted in the reappearance of these fragments, clearly demonstrating cleavage of γ-ENaC in the region proximal to the γ-inhibitory tract by TMPRSS2. As expected, the C-terminal ∼67 kDa fragment reflecting distal γ-ENaC cleavage was detectable in both wildtype and αβγ_R138A_-ENaC–expressing oocytes when TMPRSS2 was coexpressed. In these oocytes, we also detected a ∼21 kDa N-terminal fragment (Fig. 9*D*, *lower panel*), which corresponds to γ-ENaC cleaved only distally but not proximally to the γ-inhibitory tract (Fig. 9*C*). This suggests that distal γ-ENaC cleavage by TMPRSS2 may occur independently of the proximal cleavage event. In addition, we detected a ∼12 kDa N-terminal γ-ENaC fragment in both groups of oocytes coexpressing TMPRSS2 (Fig. 9*D*, *lower panel*). This fragment probably represents a degradation product of γ-ENaC, but its identity and possible function remain to be determined.

In summary, our data demonstrate that TMPRSS2 can cleave γ-ENaC at the polybasic site proximal to the γ-inhibitory tract. Thus, TMPRSS2 may play a dual role in proteolytic ENaC activation because of its ability to cleave γ-ENaC both proximally and distally to the γ-inhibitory tract.

### The arginine residue R153 at the proximal end of the γ-11 sequence may serve as an additional TMPRSS2 cleavage site in γ-ENaC

In the previous section, we demonstrated that R138 in the 135-RKRR-138 polybasic tract is not essential for TMPRSS2-dependent γ-ENaC cleavage proximal to the critical γ-11 inhibitory sequence. To investigate a possible involvement of the three remaining positively charged amino acid residues (R135, K136, and R137), we generated a mutant subunit γ_RKRR138AAAA_ (Fig. 7). Interestingly, a significant approximately twofold stimulatory effect of TMPRSS2 on ΔI_ami_ was preserved in αβγ_RKRR138AAAA_-ENaC–expressing oocytes. However, it was significantly smaller than the approximate fourfold stimulatory effect of TMPRSS2 on ΔI_ami_ in matched oocytes expressing wildtype αβγ-ENaC (Fig. 10*A*, *top and middle panels*; Fig. 10*B*). The failure of the γ_RKRR138AAAA_ mutant to prevent the stimulatory effect of TMPRSS2 suggests that an additional cleavage site exists in the region proximal to the γ-inhibitory tract. Interestingly, the first amino acid residue of the γ-11 sequence (R153) has previously been described as a plasmin cleavage site (31). We hypothesized that TMPRSS2 is also able to cleave γ-ENaC after R153, thereby increasing ΔI_ami_. Importantly, replacing R153 by an alanine in addition to replacing the four positively charged residues 135-RKRR-138 with alanines (γ_RKRR138AAAA__+__R153A_; Fig. 7) essentially abolished the stimulatory effect of TMPRSS2 on ΔI_ami_ (Fig. 10*A*, *lower panel*; 10*B*). This finding suggests that R153 can serve as an additional TMPRSS2 cleavage site in γ-ENaC. This additional cleavage site may also explain our previous observation (Fig. 8), that a moderate stimulatory effect of TMPRSS2 was preserved even when all positively charged residues distal to the γ-inhibitory tract were replaced by alanines (γ_RKRK178AAAA; K168A; K170A; R172A; K189A_-ENaC). Indeed, introducing an additional arginine-to-alanine substitution (R153A) to this mutant γ-ENaC (γ_RKRK178AAAA; K168A; K170A; R172A; K189A__+__R153A_; Fig. 7) fully abolished TMPRSS2-dependent stimulation of ΔI_ami_ (Fig. 10*C*). This finding further supports the conclusion that γ-ENaC cleavage at R153 may be accomplished by TMPRSS2 and may partially activate ENaC.

Collectively, our data obtained in the oocyte expression system demonstrate that TMPRSS2 is an ENaC-activating membrane-anchored protease, which can cleave γ-ENaC at the cell surface and intracellularly at multiple cleavage sites localized distally and proximally to the γ-inhibitory tract leading to complete proteolytic ENaC activation.

### TMPRSS2 contributes to proteolytic ENaC activation in H441 human airway epithelial cells

**Figure 11:**
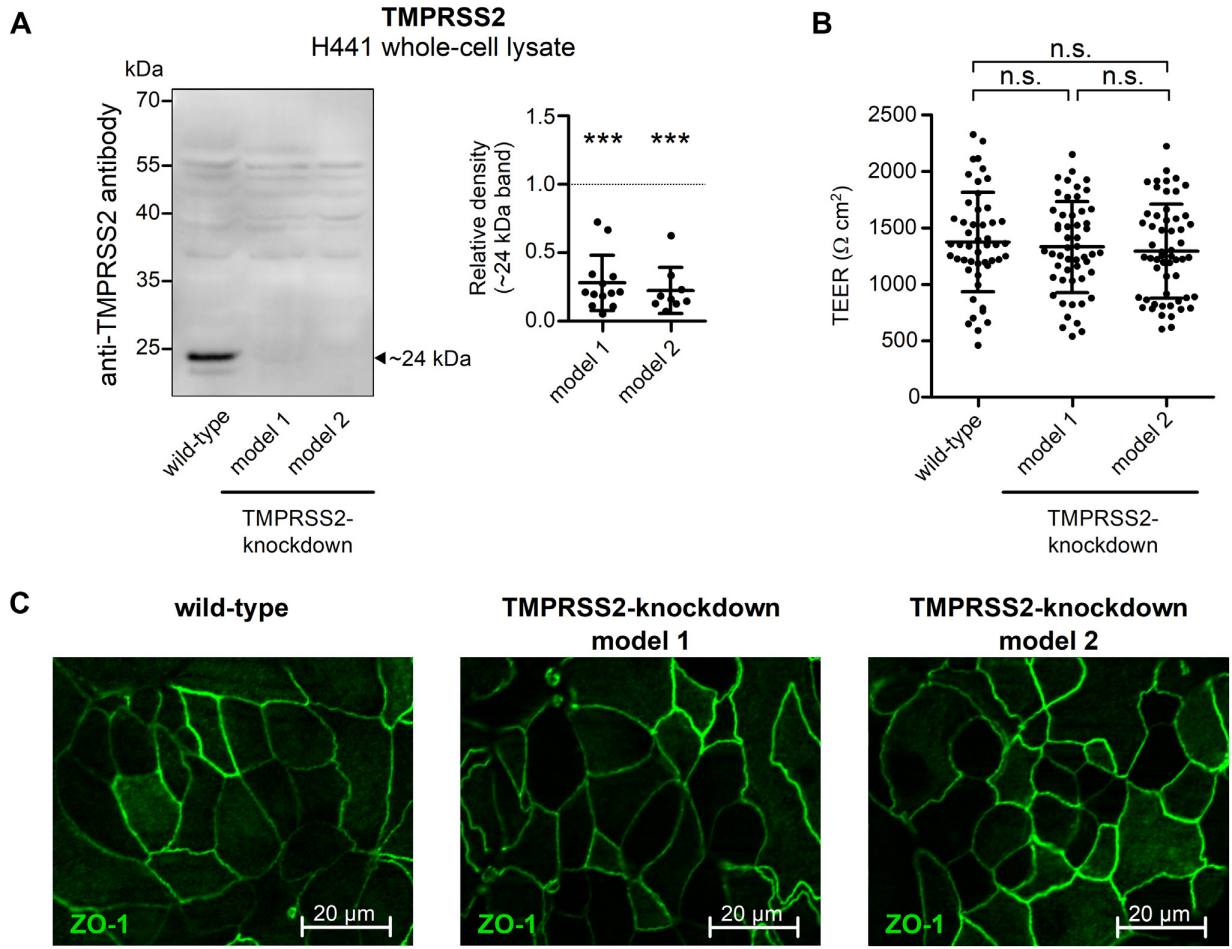
**TMPRSS2 knockdown in H441 human airway epithelial cells does not affect normal epithelial monolayer formation in culture.***A*, *left panel*, representative western blot showing endogenous expression of TMPRSS2 in H441 cells without (wildtype) or with TMPRSS2 knockdown (models 1 and 2) detected using a TMPRSS2-specific antibody. TMPRSS2 in its activated cleaved form (catalytic chain, ∼24 kDa) is indicated by a *filled arrowhead*. *Right panel*, densitometric evaluation of TMPRSS2 expression from similar blots as shown in *left panel*. In each blot, the density value of the ∼24 kDa TMPRSS2 band obtained for TMPRSS2-knockdown model 1 (n = 13) or model 2 (n = 9) was normalized to that of the corresponding TMPRSS2 band obtained in wildtype. *Dashed line* indicates a normalized density value of one (no effect). Mean ± SD and individual data points are shown; ∗∗∗*p* < 0.001; one-sample Student’s *t* test compared with wildtype (1.0). *B*, transepithelial electrical resistance (TEER) values recorded in wildtype or two different TMPRSS2-knockdown H441 cell models on day 8 after seeding cells on permeable supports. Mean ± SD and individual data points are shown; ns, one-way ANOVA with Bonferroni post hoc test (49 ≤ n ≤ 56). *C*, immunofluorescence staining for the tight junction Zonula occludens-1 protein (ZO-1, in *green*) in control or two different TMPRSS2-knockdown H441 cell models was performed on day 9 after seeding cells on permeable supports. One representative image is shown (n = 3). ns, not significant; TMPRSS2, transmembrane serine protease 2.

ENaC mediates transepithelial sodium absorption in respiratory epithelia (4, 80, 81, 82), in which TMPRSS2 is known to be abundantly expressed (57, 60, 61, 63, 83, 84). To test whether TMPRSS2 contributes to proteolytic ENaC activation in human respiratory epithelial cells, we generated two TMPRSS2-knockdown cell models based on the established H441 human distal airway epithelial cell line, which endogenously expresses ENaC and TMPRSS2 (84, 85, 86). TMPRSS2-knockdown cell models were generated by CRISPR/CRISPR-associated protein 9 (Cas9) technology using two alternative single-guide RNAs (sgRNAs) targeting exon 6 (model 1) or exon 9 (model 2) of TMPRSS2. In both models, successful TMPRSS2 knockdown was confirmed using western blot analysis of whole-cell lysates. Importantly, the expression of the TMPRSS2 catalytic domain (∼24 kDa band) was dramatically reduced in TMPRSS2-knockdown H441 cells compared with wildtype cells (Fig. 11*A*). The commercial TMPRSS2-specific antibody used in these experiments was validated using whole-cell lysates from oocytes expressing HA-labeled TMPRSS2 (Fig. S4). We also confirmed that in TMPRSS2-knockdown cells, epithelial integrity was preserved including transepithelial electrical resistance (Fig. 11*B*) and tight junction formation (Fig. 11*C*). PRSS8 (prostasin, CAP1) is critical for epithelial monolayer formation (87, 88, 89) and possibly involved in protease-mediated regulation of sodium absorption in human airway epithelia (57). To rule out a compensatory upregulation of PRSS8 in TMPRSS2 knockdown cells, we performed western blot experiments, which did not reveal a significant effect of TMPRSS2 knockdown on PRSS8 expression in model 1 and only a minor reduction of PRSS8 expression in model 2 (Fig. S5). Thus, the generated H441 cell models appeared to be suitable to study a potential regulatory role of TMPRSS2 in ENaC-mediated sodium absorption in differentiated human airway epithelial cells.

**Figure 12:**
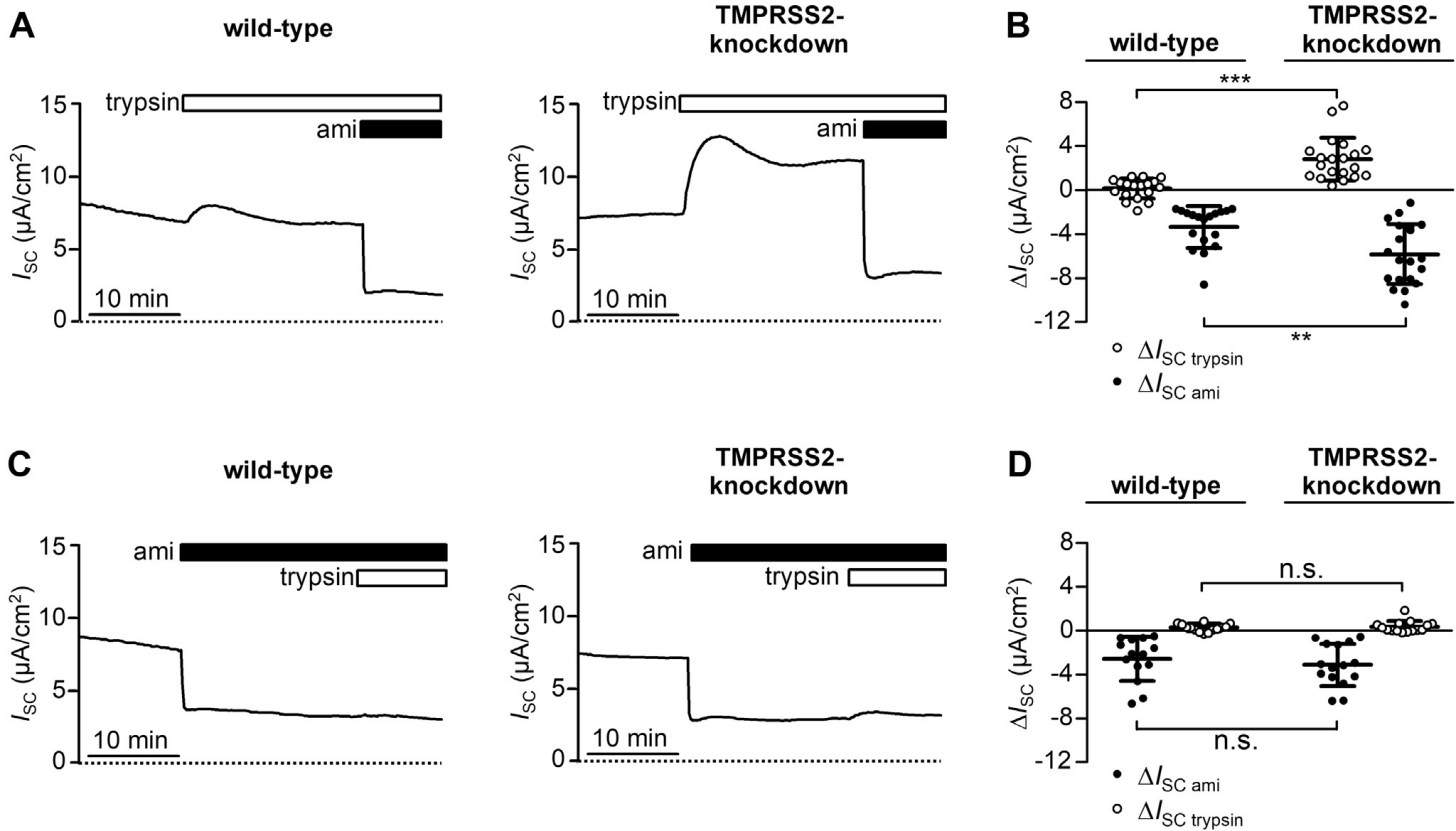
**TMPRSS2 is involved in proteolytic ENaC activation in H441 cells.***A* and *C*, representative equivalent short circuit current (*I*_SC_) recordings are shown from wildtype H441 cells (wildtype, *left traces*) or TMPRSS2-knockdown H441 cells from model 1 (TMPRSS2 knockdown, *right traces*). *B* and *D*, summary data obtained from similar experiments as shown in (*A* and *C*). Trypsin (20 μg/ml) and amiloride (ami, 10 μM) were present in the apical bath solution as indicated by *open* and *filled horizontal bars*, respectively. Initial parts of recordings (∼30 min) corresponding to the equilibration phase after transferring the cells into Ussing chambers and applying Ringer’s solution to the apical compartment are omitted for clarity. Effect of trypsin on *I*_SC_ (Δ*I*_SC__trypsin_; *open data points*) in the absence (*B*) or the presence (*D*) of apical ami determined in each individual recording by subtracting the *I*_SC_ measured before trypsin application from the current level reached in the presence of trypsin before ami application (*B*) or at the end of the recording (*D*). Effect of ami on *I*_SC_ (Δ*I*_SC ami_; *filled data points*) in the presence (*B*) or the absence (*D*) of apical trypsin was determined in each individual recording by subtracting the *I*_SC_ measured before ami application from the current level reached in the presence of ami at the end of the recording (*B*) or immediately before trypsin application (*D*). Absolute *I*_SC_ values obtained in these experiments, which were used to calculate Δ*I*_SC__trypsin_ and Δ*I*_SC ami_, are shown in Figure S6. Mean ± SD and individual data points are shown; ∗∗∗*p* < 0.001; ∗∗*p* < 0.01; ns, two-tailed Mann–Whitney test (14 ≤ n ≤ 20). ENaC, epithelial sodium channel; ns, not significant; TMPRSS2, transmembrane serine protease 2.

To assess ENaC-mediated electrogenic transepithelial sodium transport in H441 cells, we performed equivalent short-circuit current (*I*_SC_) measurements in modified Ussing chambers, which allowed stable *I*_SC_ recordings for more than 30 min as described previously (28). Representative *I*_SC_ recordings and summary data obtained from TMPRSS2-knockdown cells (model 1) and matched wildtype H441 cells are shown in Figure 12. Baseline *I*_SC_ values in TMPRSS2-knockdown H441 cells were similar to those in wildtype cells averaging 3.9 ± 4.4 μA/cm^2^ (n = 35) and 4.6 ± 4.0 μA/cm^2^ (n = 32), respectively (Fig. S6*A*). To test whether TMPRSS2 knockdown reduces proteolytic ENaC activation in H441 cells, we applied a prototypical protease trypsin to the apical bath solution. Trypsin was chosen in these experiments because its substrate specificity is similar to that of TMPRSS2 (79). Therefore, it was expected to rescue a possible incomplete proximal and distal γ-ENaC cleavage caused by TMPRSS2 knockdown. In wildtype H441 cells, apical application of trypsin only marginally affected baseline *I*_SC_ (Fig. 12*A*, *left trace*) with an average effect of 0.3 ± 0.9 μA/cm^2^ (n = 18; Figs. 12*B* and S6*B*). This confirms previous reports that under baseline conditions, ENaC localized in the apical membrane of H441 is largely cleaved by endogenous proteases and, therefore, cannot be further activated by apical application of exogenous proteases (28, 86). Importantly, a pronounced stimulatory effect of trypsin was observed in TMPRSS2-knockdown H441 cells (Fig. 12*A*, *right trace*) with an average *I*_SC_ increase of 3.0 ± 2.0 μA/cm^2^ (n = 20; Figs. 12*B*; and S6*B*). This indicates that in TMPRSS2-knockdown cells, proteolytic ENaC activation is incomplete and that channels present at the apical cell surface can be further stimulated by application of trypsin. In the presence of trypsin, subsequent application of amiloride (10 μM) caused a robust *I*_SC_ decrease, which was significantly larger in TMPRSS2-knockdown cells than in wildtype cells averaging −5.8 ± 2.8 μA/cm^2^ (n = 20) and −3.3 ± 1.9 μA/cm^2^ (n = 18), respectively (Figs. 12, *A* and *B* and S6*B*). Importantly, when trypsin was applied in the presence of amiloride, it stimulated *I*_SC_ neither in wildtype cells (Fig. 12*C*, *left trace*) nor in TMPRSS2-knockdown cells (Fig. 12*C*, *right trace*). This confirms that the prominent stimulatory effect of trypsin on *I*_SC_ observed in TMPRSS2-knockdown cells under baseline conditions is due to increased ENaC-mediated transepithelial sodium transport. Interestingly, in TMPRSS2-knockdown H441 cells, the inhibitory effect of amiloride application on baseline *I*_SC_ was similar to that in wildtype cells, averaging −3.1 ± 1.9 μA/cm^2^ (n = 15) and −2.6 ± 2.0 μA/cm^2^ (n = 14), respectively (Figs 12*D* and S6*C*). This is consistent with the finding that baseline *I*_SC_ values in TMPRSS2-knockdown H441 cells were similar to those of wildtype cells and confirms that a large portion of baseline *I*_SC_ in these cells is due to ENaC-mediated electrogenic Na^+^ transport. Using the same experimental strategy, we also investigated TMPRSS2-knockdown H441 cells from model 2 (Fig. S7), which confirmed the results obtained with model 1. As additional control, we generated a H441 cell line using CRISPR/Cas9 technique and an sgRNA targeting firefly luciferase (luc), which is not expressed in human cells (nontargeting control). As expected, TMPRSS2 expression and transepithelial electrical resistance in these luc-knockdown H441 cells were similar to those observed in wildtype cells (Fig. S8, *A* and *B*). Importantly, like in wildtype cells, application of trypsin did not significantly alter *I*_SC_ (Fig. S8, *C*–*F*). This indicates that incomplete proteolytic ENaC activation observed in TMPRSS2-knockdown cells is not caused by an unspecific effect of exposing cells to the CRISPR/Cas9 procedure.

In conclusion, TMPRSS2 knockdown results in the appearance of a population of incompletely cleaved channels at the apical surface of H441 cells as evidenced by the finding that ENaC-mediated *I*_SC_ can be further stimulated by exogenous trypsin. This indicates that endogenously expressed TMPRSS2 contributes to normal proteolytic ENaC processing and activation in H441 human airway epithelial cells.

## Discussion

In this study, we investigated the functional interaction of human αβγ-ENaC with human TMPRSS2 in the *X. laevis* oocyte expression system and in H441 human airway epithelial cells. We made the following observations: (1) coexpression of TMPRSS2 and ENaC stimulated ENaC-mediated whole-cell currents by approximately threefold, mainly because of a large increase of ENaC average open probability; (2) a catalytically inactive TMPRSS2 mutant (S441A) failed to activate ENaC, whereas inhibition of the catalytic activity of TMPRSS2 at the cell surface by aprotinin only partially reduced the stimulatory effect of TMPRSS2 on ENaC; (3) in oocytes coexpressing ENaC and TMPRSS2, fully cleaved γ-ENaC was not only present at the cell surface, but a substantial fraction of intracellular γ-ENaC was also fully cleaved; (4) the stimulatory effect of TMPRSS2 on ENaC was completely abolished by introducing a disulfide bond, to prevent the release of the γ-inhibitory tract from its binding site, and was rescued by reducing this bond with DTT; (5) TMPRSS2 cleaved γ-ENaC at multiple sites not only in the region distal but also proximal to the γ-inhibitory tract; and (6) TMPRSS2 knockdown in H441 cells led to incomplete proteolytic activation of endogenously expressed ENaC. Taken together, these findings indicate that TMPRSS2 can proteolytically activate ENaC by cleaving its γ-subunit possibly at more than one cleavage site. Importantly, TMPRSS2 accomplishes γ-ENaC cleavage not only at the cell surface but also intracellularly, including the critical final cleavage step at the distal cleavage site. Thus, in oocytes coexpressing TMPRSS2 and ENaC, a substantial portion of channels reaching the plasma membrane are fully cleaved and active. Moreover, our findings in H441 airway epithelial cells provide proof of principle that TMPRSS2 can play a role in proteolytic ENaC activation not only in the oocyte expression system but also in polarized epithelial cells. Thus, TMPRSS2 is a likely candidate protease involved in proteolytic ENaC regulation *in vivo*.

The strong stimulatory effect of TMPRSS2 on ENaC observed in the present study is in good agreement with a previous report demonstrating that coexpression of human TMPRSS2 and rat ENaC stimulated ENaC-mediated currents by ∼2.6-fold (46). Our experiments with MTSET and the mutant αβ^S520C^γ-ENaC revealed that TMPRSS2 coexpression increased average *P*_O_ of ENaC to an extent that can fully explain the observed stimulatory effect of TMPRSS2 on ENaC whole-cell currents. Interestingly, in the first study, in which the effect of TMPRSS2 on ENaC was investigated, ENaC-mediated currents and ENaC protein levels were found to be dramatically reduced in oocytes coexpressing rat ENaC and human TMPRSS2 (57). This was thought to be due to enhanced proteolytic degradation of ENaC by TMPRSS2 (57). Our biotinylation experiments suggested that the amount of γ-ENaC detected at the cell surface was lower in oocytes coexpressing ENaC and TMPRSS2 than in oocytes expressing ENaC alone. However, we demonstrated that MTSET application to oocytes expressing αβ^S520C^γ-ENaC alone increased ENaC currents to a level similar to that observed in oocytes coexpressing TMPRSS2 and αβ^S520C^γ-ENaC. In these latter oocytes, MTSET had no further stimulatory effect. This indicates that after treatment with MTSET, the number of fully active channels present at the cell surface of oocytes expressing αβ^S520C^γ-ENaC alone was similar to that in oocytes coexpressing αβ^S520C^γ-ENaC and TMPRSS2. Thus, we have no indication that under our experimental conditions, TMPRSS2 reduced ENaC expression at the cell surface. Nevertheless, we observed additional small intracellular C- and N-terminal γ-ENaC cleavage fragments in oocytes coexpressing ENaC with TMPRSS2. These fragments were not detected at the cell surface and, therefore, may represent intracellular γ-ENaC degradation products consistent with the idea that TMPRSS2 may contribute to intracellular ENaC degradation under certain conditions. Interestingly, TMPRSS4 (CAP2) has been reported to produce a similar C-terminal γ-ENaC fragment probably by cleaving at a highly conserved arginine residue (R515) in the region preceding the second transmembrane domain of γ-ENaC. Whether this C-terminal γ-ENaC fragment is a degradation product or has a functional role is unclear (40). In the present study, we did not further investigate the nature and possible function of the short γ-ENaC cleavage fragments detected in oocytes coexpressing TMPRSS2.

In parallel with our current measurements, we used an established fluorogenic substrate assay (56) to investigate trypsin-like proteolytic activity at the cell surface of oocytes. Using this assay, we demonstrated strong trypsin-like proteolytic activity at the cell surface of oocytes expressing TMPRSS2, which was not observed in control oocytes or in oocytes expressing a catalytically inactive mutant TMPRSS2 (S441A). This latter mutant failed to stimulate ENaC currents, which indicates that the proteolytic activity of TMPRSS2 is essential for its stimulatory effect on ENaC. In contrast to these findings, we previously observed in similar coexpression experiments that both wildtype PRSS8 and catalytically inactive mutant PRSS8–S238A induced strong proteolytic activity at the cell surface and caused maximal proteolytic ENaC activation (56). Thus, unlike for TMPRSS2, the proteolytic activity of PRSS8 is not essential for its stimulatory effect on ENaC, which is consistent with previously reported findings (22, 45, 55). This suggests that both PRSS8 and PRSS8–S238A activate ENaC indirectly possibly by activating and/or recruiting endogenous trypsin-like proteases, which probably cleave the channel at the plasma membrane (56). In contrast, TMPRSS2 is likely to cause proteolytic channel activation by directly cleaving ENaC. However, proteolytic ENaC activation by TMPRSS2 is not dependent on catalytic activity of TMPRSS2 at the plasma membrane. Indeed, the stimulatory effect of TMPRSS2 on ENaC is preserved to a large extent when cell surface activity of TMPRSS2 is inhibited by aprotinin. Importantly, our western blot data demonstrated fully cleaved γ-ENaC in the intracellular protein fraction. Taken together, this indicates that TMPRSS2 can fully cleave γ-ENaC not only at the cell surface but also intracellularly before the ion channel reaches the plasma membrane. This finding argues against the currently accepted paradigm that ENaC has to be trafficked to the plasma membrane to be fully cleaved by membrane-anchored proteases (14, 21). We are not aware of any studies demonstrating that another membrane-anchored serine protease can perform the final γ-ENaC cleavage intracellularly like TMPRSS2. Nevertheless, it is conceivable that this is not a unique feature of TMPRSS2 but may be a more general property shared by other members of the membrane-anchored serine protease family. Indeed, the stimulatory effect of matriptase (CAP3) on ENaC was aprotinin resistant (51). Given that aprotinin has a potent inhibitory effect on matriptase (90), this observation provides indirect evidence that matriptase may also activate ENaC intracellularly. In contrast, aprotinin prevented the stimulatory effects of coexpressed PRSS8 or TMPRSS4 (CAP2) on ENaC. This indicates that the stimulatory effect of these latter proteases requires the presence of proteolytic activity at the cell surface (40, 43, 50, 51). It is tempting to speculate that distinct properties of membrane-anchored proteases and their mix in a particular tissue or cell type will determine whether ENaC is preferentially cleaved at the plasma membrane or in intracellular compartments. Indeed, aprotinin has been reported to inhibit ENaC-mediated sodium transport to different degrees in toad urinary bladder (91), A6 *Xenopus* kidney epithelial cells (43), mpkCCD_c14_ mouse cortical collecting duct cells (50), and primary nasal epithelial cells (57). Intriguingly, aprotinin treatment resulting in high urinary aprotinin concentrations failed to prevent basal proteolytic ENaC cleavage in kidney of healthy mice (92). This suggests predominant intracellular proteolytic ENaC activation in the distal nephron under physiological conditions consistent with previously reported findings that ENaC is present mainly in its cleaved form in the apical membrane of mouse and rat distal nephron (93). However, it is conceivable that aprotinin-insensitive proteases also contribute to proteolytic ENaC activation in the distal nephron, which complicates the unambiguous interpretation of these experiments. Future studies are needed to explore the subcellular distribution of TMPRSS2 and its colocalization with ENaC in intracellular organelles in different epithelial tissues.

Using site-directed mutagenesis (γ_S155C;Q426C_) based on the analysis of the recently published cryo-EM structures of ENaC (7, 8), we demonstrated that the stimulatory effect of TMPRSS2 on ENaC was fully abolished by introducing a disulfide bond between the γ-inhibitory tract and its binding site. Importantly, reducing this bond by DTT resulted in complete rescue of the stimulatory effect of TMPRSS2 on ENaC. This finding clearly shows that proteolytic activation of ENaC by TMPRSS2 not only requires dual cleavage of γ-ENaC but also critically depends on the subsequent release of the γ-inhibitory tract from its binding site. In oocytes coexpressing αβγ_S155C;Q426C_-ENaC and TMPRSS2, proteolytic cleavage of the γ-subunit is complete. However, the excised γ-inhibitory tract remains tightly attached to its binding site, unless the disulfide bond is reduced by DTT resulting in the release of the γ-inhibitory tract and channel activation. This hypothesis is consistent with the recently published cryo-EM structure of human ENaC demonstrating extensive contacts of the γ-inhibitory tract with the finger and thumb domains (8). The final distal cleavage of γ-ENaC is required to destabilize these interactions. The conformational changes induced by proteolytic cleavage of γ-ENaC and associated with the release of the γ-inhibitory tract are currently unknown but may involve rearrangements of GRIP, thumb, and finger domains (7). Our novel experimental approach to uncouple cleavage of γ-ENaC from channel activation by a disulfide bond preventing the release of the γ-inhibitory tract may provide a useful tool for future studies to further elucidate the functional importance of γ-ENaC cleavage *in vitro* and *in vivo*.

The pivotal final cleavage of γ-ENaC occurs in the region distal to the γ-inhibitory tract (14, 22, 23). It has been shown that many extracellular and membrane-anchored proteases cleave γ-ENaC at distinct sites in this region (22, 24, 28, 31, 40, 41, 42). A prominent feature of this region is the highly conserved polybasic RKRK tract, known as putative prostasin (PRSS8) cleavage site. Several reports demonstrated the critical importance of the intact RKRK sequence for PRSS8-mediated stimulation of ENaC (22, 93). In contrast, we have shown that mutating the RKRK site alone did not significantly reduce the stimulatory effect of TMPRSS2 on ENaC, indicating that the intact RKRK sequence is not essential for TMPRSS2-dependent ENaC activation. However, TMPRSS2 was able to cleave at the RKRK site and substantially stimulate ENaC, when all other positively charged residues in the region distal to the γ-inhibitory tract were substituted by alanines and only the RKRK tract was left intact. Finally, replacing all lysines and arginines by alanines in this γ-ENaC region significantly decreased proteolytic stimulation of ENaC by TMPRSS2. Thus, these results suggest that TMPRSS2 has less stringent substrate specificity than PRSS8. This property of TMPRSS2 resembles that of trypsin or matriptase (CAP3), which also cleave γ-ENaC at multiple positions, and extensive mutations are required to block their stimulatory effects on ENaC currents (28, 41).

Our computer simulations suggest that two arginine residues (R172 or R178) are preferential cleavage sites of TMPRSS2 in γ-ENaC. This prediction corresponds well with the reported optimal TMPRSS2 cleavage sequence identified using short peptide screening analysis (62). According to Lucas *et al.* (62), TMPRSS2 strongly prefers substrates with an arginine (R) over those with a lysine (K) residue at P1 position. The P2–P4 specificity of TMPRSS2 is less stringent. Threonine, phenylalanine, tryptophan, alanine, or valine (T, F, W, A, or V) at P2; glutamate, methionine, or glutamine (E, M, or Q) at P3; and glycine, isoleucine, or methionine (G, I, or M) at P4 position are preferred, but almost all other amino acid residues (with few exceptions) can also be tolerated at these positions (62). Thus, both regions in γ-ENaC (169-GKAR-172 and 175-FTGR-178) may be cleaved by TMPRSS2 with similar efficiency. Interestingly, a lysine residue (K) at position P2 is not compatible with TMPRSS2 cleavage (62), which suggests that TMPRSS2 may not cleave after arginine residue R180 in the sequence 177-GRKR-180. To conclude, R172 and R178 are the most probable TMPRSS2 cleavage sites in the region distal to the γ-inhibitory tract. In addition, our functional data indicate that TMPRSS2 may also cleave after R153. This arginine residue has been identified in a previous study as a putative plasmin cleavage site (31). However, the putative cleavage of γ-ENaC at R153 did not result in complete stimulation of ENaC-mediated currents, indicating that this cleavage site is functionally less important than the more distal cleavage sites R172 and R178.

Our experiments with the furin-resistant mutant ENaC (γ_R138A_) indicated that TMPRSS2 may cleave γ-ENaC in the preceding 135-RKR-137 sequence. According to the reported TMPRSS2 substrate specificity (62), R135 is the most probable cleavage site within this sequence. Thus, because of a less stringent substrate specificity than furin, TMPRSS2 may also contribute to the intracellular cleavage of γ-ENaC in the region proximal to the γ-inhibitory tract. The ability to perform the proximal cleavage of γ-ENaC has also been shown for matriptase (CAP3) (41) and TMPRSS4 (CAP2) (42). It is tempting to speculate that TMPRSS2 and possibly other membrane-anchored proteases may also cleave α-ENaC at polybasic tracts flanking the α-inhibitory tract, along with furin or furin-like convertases (14, 21). However, the hypothesis that membrane-anchored serine proteases like TMPRSS2 may play such a universal role in proteolytic ENaC activation under physiological conditions remains to be confirmed experimentally.

It is well established that TMPRSS2 plays an important pathophysiological role in respiratory lung diseases by promoting cellular entry of several viruses, including influenza A and B virus, severe acute respiratory syndrome–associated coronavirus (SARS-CoV), Middle East respiratory syndrome–associated coronavirus (MERS-CoV) (94), and the novel severe acute respiratory syndrome–associated coronavirus 2 (SARS-CoV-2) (95). Conversely, ENaC-mediated transepithelial sodium transport may be of critical importance in the pathophysiology of airway infection including coronavirus disease 2019 (COVID-19) (96). Interestingly, *Tmprss2* knockout mice have no obvious phenotype under baseline conditions (97). It remains to be seen whether a functional relevance of TMPRSS2-dependent ENaC activation becomes evident in challenging conditions where increased alveolar or renal ENaC activity is needed, for example, in pulmonary edema or salt deprivation, respectively. However, the physiological role of TMPRSS2 *in vivo* presently remains elusive.

Nevertheless, our finding that proteolytic ENaC activation is significantly reduced in TMPRSS2-knockdown H441 cells makes TMPRSS2 a likely candidate to be involved in proteolytic ENaC activation in human distal airway epithelial cells under physiological conditions. Baseline ENaC-mediated *I*_SC_ in TMPRSS2-knockdown cells was similar to that in H441 control cells but could be further stimulated by application of trypsin. This stimulatory effect of trypsin was not observed in H441 control cells and indicates that in TMPRSS2-knockdown cells, a channel population exists in the apical membrane, which is only partially cleaved. This supports the conclusion that under normal conditions, TMPRSS2 contributes to proteolytic ENaC activation in H441 cells. Despite incomplete proteolytic ENaC activation, TMPRSS2-knockdown cells attained a baseline *I*_SC_ similar to that of control cells possibly because of compensatory upregulation of ENaC-mediated transepithelial transport by other mechanisms. These were not addressed in the present study but may be of interest to be explored in future experiments. Importantly, the ability of the TMPRSS2-knockdown cells to generate a normal baseline *I*_SC_ suggests that TMPRSS2 knockdown does not severely compromise overall transepithelial ion transport function and epithelial integrity in H441 cells.

It should be noted that our transepithelial *I*_SC_ recordings in H441 cells were performed under conditions of apical surface layer (ASL) expansion because of the presence of apical bath solution in the Ussing chambers. It has been reported that ASL expansion stimulates proteolytic ENaC processing in human airway epithelial cells probably by diluting protease inhibitors (98, 99). This mechanism may serve as feedback regulation to normalize ASL by stimulating ENaC-dependent transepithelial salt and fluid absorption. On the other hand, excess proteolytic activity at the cell surface may contribute to ENaC hyperactivity and mucus dehydration in cystic fibrosis airways (80, 82). A previous study highlighted the importance of PRSS8 for ENaC-mediated alveolar fluid clearance and lung fluid balance (54). Therefore, it may be of interest to explore a possible interaction of PRSS8 and TMPRSS2 in respiratory epithelia in the context of ENaC regulation. TMPRSS2 may also be involved in proteolytic ENaC regulation in other epithelial tissues, including distal colon and aldosterone-sensitive distal nephron, where both proteins have been shown to be coexpressed (46, 64, 65). Future studies are needed to investigate the physiological role of TMPRSS2 in maintaining ASL homeostasis and in regulating ENaC function in various epithelia.

In conclusion, we demonstrated that TMPRSS2 can proteolytically activate ENaC by cleaving the channel’s γ-subunit at multiple sites resulting in the release of the γ-inhibitory tract. This critically depends on the catalytic activity of TMPRSS2 but not necessarily on its activity at the cell surface. Thus, proteolytic ENaC activation by TMPRSS2 probably occurs also intracellularly before the channel reaches the plasma membrane. Moreover, the present study suggests an involvement of TMPRSS2 in proteolytic ENaC regulation in airway epithelial cells and possibly other epithelial tissues.

## Experimental procedures

### Chemicals

Amiloride, aprotinin, DTT, α-chymotrypsin type II, and trypsin from bovine pancreas were purchased from Sigma–Aldrich. The sulfhydryl reagent MTSET was obtained from Biotium.

### Plasmids

Full-length complementary DNAs (cDNAs) encoding human α-ENaC, β-ENaC, and γ-ENaC were kindly provided by H. Cuppens. Full-length cDNA encoding human TMPRSS2 was obtained from Source BioScience UK Limited (I.M.A.G.E. Clone ID: 6199625) (100). cDNAs were subcloned into the pGEM-HE vector for experiments shown in Figure 5 or into pTLN vector for all other experiments (101). Plasmids were linearized and used as templates for cRNA synthesis using T7 (for pGEM-HE constructs) or SP6 (for pTLN constructs) RNA polymerase (mMessage mMachine; Ambion). QuikChange lightning site-directed mutagenesis kit (Agilent Technologies) was used to generate β-ENaC, γ-ENaC, and TMPRSS2 mutants and to attach an N-terminal V5 tag (GKPIPNPLLGLDST) to γ-ENaC and a C-terminal HA tag (YPYDVPDYA) to TMPRSS2. Sequences were routinely confirmed by sequence analysis (LGC Genomics).

### Isolation of oocytes and two-electrode voltage-clamp experiments

Isolation of oocytes and two-electrode voltage-clamp experiments were essentially performed as described previously (70, 102). Defolliculated stage V–VI oocytes were obtained from adult female *X. laevis* in accordance with the principles of German legislation, with approval by the animal welfare officer for the University of Erlangen-Nürnberg, and under the governance of the state veterinary health inspectorate (approval number: Az. 55.2-2532-2-527). Animals were anesthetized in 0.2% MS222 (Sigma–Aldrich), and ovarian lobes were obtained by a small abdominal incision. Oocytes were isolated from ovarian lobes using a type-2 collagenase from *Clostridium histolyticum* (Sigma–Aldrich). Oocytes were injected with the same amount (0.1 ng, unless stated otherwise) of cRNA per ENaC subunit (α, β, and γ) per oocyte. Unless stated otherwise, 2 ng of human TMPRSS2 cRNA were injected. After cRNA injection, oocytes were kept in a low sodium ND9 solution (composition in millimolar: 9 NaCl, 2 KCl, 87 *N*-methyl-d-glutamine-Cl, 1.8 CaCl_2_, 1 MgCl_2_, 5 Hepes, and pH 7.4 adjusted with Tris) supplemented with 100 units/ml sodium penicillin and 100 μg/ml streptomycin sulphate. Oocytes were studied 48 h after cRNA injection. Bath solution exchanges with a gravity-fed system were controlled by a magnetic valve system (ALA BPS-8; ALA Scientific Instruments) in combination with a TIB14 interface (HEKA). An individual oocyte was placed in an experimental chamber with a narrow flow channel (length: 45 mm; height: 3 mm; and width: 3 mm) with a U-shaped cross section of ∼8 mm^2^. The oocyte was positioned in the experimental chamber close to the site of solution inflow and was held in place by the impaling microelectrodes. To achieve rapid and reproducible solution exchanges at the oocyte, the perfusion rate was carefully adjusted for each experimental solution to ∼10 ml/min, resulting in a flow velocity of ∼20 mm/s. The flow channel drained into a reservoir (2 cm × 1 cm) from which the solution was continuously removed *via* a suction tube. The suction tube was adjusted to maintain the fluid level in the flow channel at ∼2 mm. Oocytes were clamped at a holding potential of –60 mV using an OC-725C amplifier (Warner Instruments) connected by an LIH-1600 (HEKA) to a personal computer. Pulse 8.78 software (HEKA) was used for data acquisition. Whole-cell current recordings started typically within 10 s after impalement of an oocyte with microelectrodes. ENaC-mediated whole-cell currents (ΔI_ami_) were determined by washing out amiloride (2 μM) with amiloride-free bath solution and subtracting the whole-cell currents measured in the presence of amiloride from the corresponding whole-cell currents recorded in its absence. ND96 solution was used as standard bath solution (composition in millimolar: 96 NaCl, 2 KCl, 1.8 CaCl_2_, 1 MgCl_2_, 5 Hepes, and pH 7.4 adjusted with Tris).

### Cell surface protein detection and western blot analysis in oocytes

To separate cell surface proteins from intracellular proteins, a biotinylation approach was used essentially as described previously (23, 56, 70, 103). Western blot analysis was performed under reducing conditions. Human TMPRSS2 with a C-terminal HA tag was detected using a rat monoclonal anti-HA antibody (clone 3F10; Roche Diagnostics) at a dilution of 1:1000 and a secondary horseradish peroxidase–conjugated goat antirat antibody (AffiniPure; Jackson Immunoresearch) at a dilution of 1:10,000. C-terminal cleavage fragments of human γ-ENaC were detected using a subunit-specific antibody generated in rabbits against human γ-ENaC (Pineda Antibody Service) (9) at a dilution of 1:5000 and horseradish peroxidase–labeled secondary goat anti-rabbit antibodies (Santa Cruz Biotechnology) at a dilution of 1:50,000. To validate separation of cell surface proteins from intracellular proteins by biotinylation, blots were stripped and reprobed using a polyclonal rabbit anti-β-actin antiserum (Sigma–Aldrich) at a dilution of 1:5000.

In some experiments, cleavage fragments of γ-ENaC were analyzed in whole-cell lysates prepared essentially as described previously (70). The antibodies used for the detection of C-terminal γ-ENaC cleavage fragments were the same as described previously. N-terminal cleavage fragments of the V5-tagged γ-ENaC were detected using mouse monoclonal anti-V5 antibody (Invitrogen) at a dilution of 1:1000 and a secondary horseradish peroxidase–labeled goat antimouse antibody (Abcam) at a dilution of 1:50,000. Densitometry was performed using ImageJ (National Institutes of Health). ATX Ponceau S (Fluka) membrane staining was used to control protein loading.

### Determination of TMPRSS2 proteolytic activity at the surface of oocytes

TMPRSS2-dependent proteolytic activity at the cell surface was quantified using the fluorogenic substrate Boc–QAR–AMC and the experimental protocol previously established in our laboratory for the detection of trypsin-like proteolytic activity at the surface of oocytes expressing mouse PRSS8 (56). Each individual oocyte was placed into a well of a 96-well plate containing 100 μl of standard ND9 solution supplemented with 10 μmol/l fluorogenic substrate. The fluorescence signal (360 nm excitation/465 nm emission wavelength) resulting from substrate hydrolysis at the cell surface was continuously recorded over a period up to 190 min using a TECAN GENios plate reader (Tecan).

### Homology model of human TMPRSS2 and molecular docking approach

The homology model of the catalytic domain of human TMPRSS2 (amino acid residues from 256 to 492) was built using the default modeling in the automodel-class suite of MODELLER 9.12 package (Ben Webb, San Francisco, CA) (104, 105) and the crystal structure of the catalytic domain of human hepsin (Protein Data Bank accession no.: 1Z8G) as a template (106, 107). The catalytic domain of hepsin has ∼41% identical and ∼57% similar amino acid residues compared with TMPRSS2 and therefore was suitable for TMPRSS2 homology modeling as a template. The sequence alignment of hepsin and TMPRSS2 was performed using T-Coffee Expresso package (http://www.tcoffee.org) (108, 109). TMPRSS2 homology models generated using MODELLER were ranked by DOPE and GA341 scores. The top ranked model was refined using GalaxyRefine server and verified using the PROCHECK, ERRAT, and Verify3D algorithms (110, 111, 112, 113, 114). The structures of three 6-mer peptides corresponding to three segments of the γ-ENaC sequence (169-GKARDF-174, 177-GRKRKF-182, or 187-IHKASN-192) were generated using UCSF Chimera developed by the Resource for Biocomputing, Visualization, and Informatics at the University of California, San Francisco, with support from National Institutes of Health P41-GM103311 (115). The length of the simulated peptides was limited to six (6-mer peptides) to improve the docking accuracy and to take into account the typical length of a protease recognition motif in a substrate, which typically includes only a few amino acid residues proximal and distal to the scissile peptide bond (79). Putative binding modes of these peptides within the catalytic domain of TMPRSS2 were predicted using the molecular docking software AutoDock Vina (The Scripps Research Institute, La Jolla, CA) (116). Peptides and TMPRSS2 were prepared for docking using AutoDockTools 1.5.6 (117, 118). The docking grid box with the dimensions of 40 × 25 × 25 Å and the center close to TMPRSS2 catalytic triad was assigned using AutoDockTools 1.5.6. Ten runs of the program were performed with nine binding modes generated per run, giving a total number of 90 docked binding modes per peptide. Docking modes were inspected, evaluated, and visualized using UCSF Chimera. TMPRSS2 molecular surface calculation and electrostatic coloring was performed using the MSMS, PDB2PQR, and APBS packages (119, 120, 121).

### H441 cell culture experiments

The National Cancer Institute-H441 (H441) (American Type Culture Collection HTB-174) human lung epithelial cell line was obtained from American Type Culture Collection and used as wildtype control. Two TMPRSS2-knockdown H441 cell lines and a luc-knockdown H441 cell line (nontargeting control) were generated from wildtype cells by lentiviral transduction using pLenti-CRISPR-V2–based lentiviral particles targeting TMPRSS2 or firefly luciferase (luc). The latter is not expressed in human cells. Two different sgRNA sequences (#1: ACTGGAACGAGAACTACGGG; #2: GTCCAGAACGTCCACGTGTG) targeting exon 6 or exon 9 of human TMPRSS2, respectively, were designed using GPP sgRNA Designer (https://portals.broadinstitute.org/gpp/public/analysis-tools/sgrna-design) and cloned into the pLenti-CRISPR-V2 vector as described previously (122, 123). The following sgRNA sequence was used to generate luc-knockdown H441 cells: TACAAACGCTCTCATCGACA. pLenti-CRISPR-V2 was a gift from Feng Zhang (Addgene plasmid #52961; http://n2t.net/addgene/52961; Research Resource Identifier: Addgene 52961). To generate CRISPR/Cas9-V2-based lentiviral particles targeting TMPRSS2 or luc, human embryonic kidney 293T cells were transfected with vesicular stomatitis virus glycoprotein expression plasmid (pVSV-G), the HIV gag/pol packaging plasmid pCMVΔR8.9, and lentiviral vector pLenti-CRISPR-V2 encoding sgRNAs targeting TMPRSS2 or luc at a mass ratio of 1:2:2 using calcium phosphate. Cell-culture supernatants were collected 48 hpi, passed through 0.45 μm pore size filters, and concentrated by centrifugation through size-exclusion filters (Amicon, Millipore) and stored at −80 °C. Three days postinfection, transduced H441 cells were selected using 2.5 μg/ml puromycin containing medium. Wildtype (passages 64–72), luc-knockdown cells (passages 66–69), and TMPRSS2-knockdown H441 cells (passages 66–74) were cultured in parallel as described previously (28). Cells were maintained in a 5% CO_2_ atmosphere at 37 °C in H441 growth medium (RPMI1640 medium [Roswell Park Memorial Institute; Biochrom], 10% fetal bovine serum, 2 mM l-glutamine, 5 μg/ml apotransferrin, 5 μg/ml insulin, 10 nM sodium selenite, 1 mM sodium pyruvate, 100 U/ml penicillin, and 10 μg/ml streptomycin). This medium was in addition supplemented with 0.5 μg/ml puromycin for TMPRSS2- and luc-knockdown H441 cells.

For transepithelial measurements, cells were cultured on permeable supports (Millicell PCF membrane inserts; Merck–Millipore) in H441 growth medium. At day 4 after seeding, this medium was replaced in the basolateral compartment by a differentiation H441 medium (RPMI1640 medium, 4% charcoal-stripped serum, 1 nM triiodothyronine, 50 nM dexamethasone, 5 μg/ml apotransferrin, 5 μg/ml insulin, and 10 nM sodium selenite). The apical side of the epithelial monolayer was kept at an air–liquid interface. Epithelial monolayer formation was controlled by measuring the transepithelial electrical resistance as previously reported (124). For these measurements, a Ringer’s solution (composition in millimolar: 117 NaCl, 25 NaHCO_3_, 4.7 KCl, 1.2 MgSO_4_, 1.2 KH_2_PO_4_, 2.5 CaCl_2_, 11 d-glucose, equilibrated to pH 7.4 with a 5% CO_2_ atmosphere) was added to the apical compartment. After 5 days at the air–liquid interface, the monolayers were transferred into Ussing chambers to measure the equivalent short circuit current (*I*_SC_) essentially as described previously (28, 124). To record the transepithelial voltage and resistance, Ringer’s solution was added to the apical compartment, and cells were allowed to equilibrate for 30 min. Stock solutions of trypsin (2 mg/ml) or amiloride (1 mM) were added directly to the apical bath solution to achieve the final concentration of 20 μg/ml or 10 μM, respectively.

Whole-cell lysates of H441 cells were obtained by scraping cells from their permeable supports and transferring them into a lysis buffer (50 mM Hepes, 150 mM NaCl, 10% glycerol, and 1% Triton X-100) supplemented with protease inhibitor mixture (“cOmplete EDTA-free” protease inhibitor mixture tablets; Roche Diagnostics). The lysates were sonicated and centrifuged for 10 min at 1000*g* to remove cell debris. Samples were boiled for 5 min at 95 °C and subjected to 10% SDS-PAGE using 25 μg of total protein per lane. Western blot analysis was performed under reducing conditions. After separation, proteins were transferred to polyvinylidene difluoride membranes by semidry electroblotting and probed with mouse monoclonal antibody against serine protease domain of human TMPRSS2 (clone P5H9-A3; catalog no.: MABF2158; lot no.: 3110143; EMD Millipore Corp) at a dilution of 1:2500 or mouse antihuman PRSS8 antibody (catalog no.: 612173; BD Transduction Laboratories) at a dilution of 1:250. Goat anti-mouse horseradish peroxidase–labeled antibody (catalog no.: ab97023; Abcam) at a dilution of 1:50,000 was used as a secondary antibody. For immunofluorescence staining experiments, H441 cells were fixed on permeable supports with 4% paraformaldehyde in PBS, permeabilized with 0.1% Triton X-100, and blocked with Roti Immunoblock (Carl Roth). These cell preparations were stained by overnight incubation with mouse monoclonal anti-ZO-1 (zonula occludens-1) antibody (clone ZO1-1A12; Invitrogen), at a dilution of 1:250 dissolved in PBS, supplemented with 0.5% bovine serum albumin, and 0.04% sodium azide. This was followed by an incubation for 1 h with a goat antimouse DyLight488 antibody (Thermo Fisher Scientific) at a dilution of 1:400. After staining, the cell preparations were mounted on slides using Dako Glycergel Mounting Medium (Agilent), and images were acquired using a ZEISS microscope (Axiovert 200M) and apotome technology.

### Statistical methods

Data are presented as mean ± SD. Normal distribution of data was assessed using D’Agostino–Pearson omnibus test. Statistical significance was assessed by an appropriate parametric test: ANOVA (with Bonferroni post hoc test) or Student’s *t* test or by a nonparametric test: Kruskal–Wallis (with Dunn’s post hoc test) or Mann–Whitney test as indicated. N indicates the number of different batches of oocytes, and *n* indicates the number of individual oocytes studied per experimental group. Statistical analysis was performed using GraphPad Prism, version 5.04 (GraphPad Software, Inc).

## Data availability

All data are contained within the article.

## Supporting information

This article contains supporting information.

## Conflict of interest

The authors declare that they have no conflicts of interest with the contents of this article.
