# Supplementary material for: Transmembrane serine protease 2 (TMPRSS2) proteolytically activates the epithelial sodium channel (ENaC) by cleaving the channel’s γ-subunit
Source: J Biol Chem. 2022 Apr 30;298(6):102004. doi: 10.1016/j.jbc.2022.102004 (PMC9163703; doi:10.1016/j.jbc.2022.102004)
Supplement: Supplemental Figures S1–S8 [file mmc1.pdf]

Transmembrane serine protease 2 (TMPRSS2) proteolytically activates the epithelial sodium channel (ENaC) by cleaving the channel's γ-subunit

Florian Sure[1], Marko Bertog[1], Sara Afonso[1], Alexei Diakov[1], Ralf Rinke[1], M. Gregor Madej[2], Sabine Wittmann[3], Thomas Gramberg[3], Christoph Korbmacher[1]* and Alexandr V. Ilyaskin[1]

Supporting information Table of Contents

Figure S1; Figure S2; Figure S3; Figure S4; Figure S5; Figure S6; Figure S7; Figure S8.

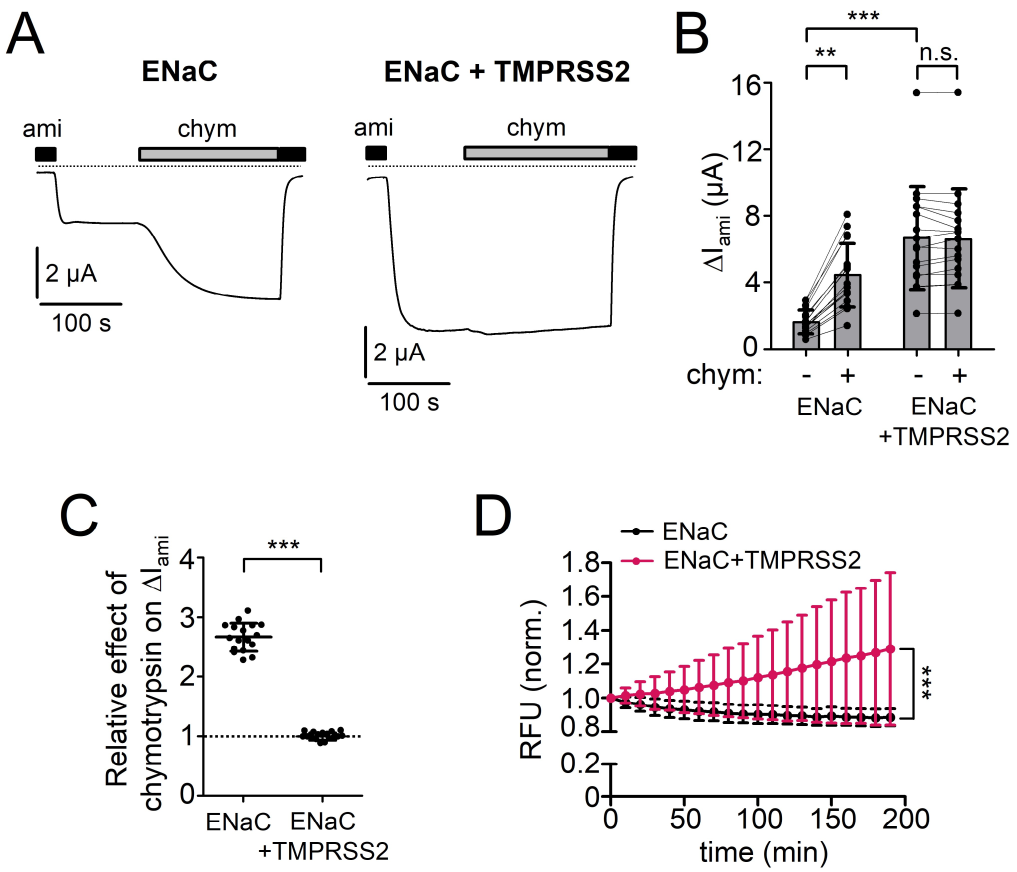

Figure S1. TMPRSS2 without C-terminal HA-tag also proteolytically activates αβγ-ENaC. (*A*) representative whole-cell current traces recorded in a human αβγ-ENaC expressing oocyte without (*left trace*, ENaC) or with (*right trace*, ENaC+TMPRSS2) human TMPRSS2 coexpression. In these control experiments a TMPRSS2 construct was used without HA-tag attached to its C-terminus. Amiloride (ami, 2 µM) and chymotrypsin (chym, 2 µg/ml) were present in the bath solution as indicated by black and grey bars, respectively. Dashed lines indicate zero current level. (*B*) ENaC-mediated amiloride-sensitive whole-cell currents (ΔI_{ami}) were determined as described in Fig. 1B from similar experiments as shown in (*A*). Lines connect data points obtained in an individual oocyte. Mean ± SD and data points for individual oocytes are shown; ***$p < 0.001$; **$p < 0.01$; n.s., not significant, Kruskall-Wallis with Dunn's post hoc test (n=17, N=3). (*C*) relative stimulatory effect of chymotrypsin on ΔI_{ami} summarized from data shown in (*B*). Dashed line indicates a normalized ΔI_{ami} value of one (no effect). Mean ± SD and data points for individual oocytes are shown; ***$p < 0.001$; two-tailed unpaired Student's *t* test. (*D*) In parallel experiments to those shown in (*A-C*), trypsin-like proteolytic activity at the cell surface (RFU = relative fluorescent unit; mean ± SD) was detected in these batches of oocytes as described in Fig. 1D. ***$p < 0.001$; two-tailed Mann-Whitney test (at the time point 190 min; n=22, N=3).

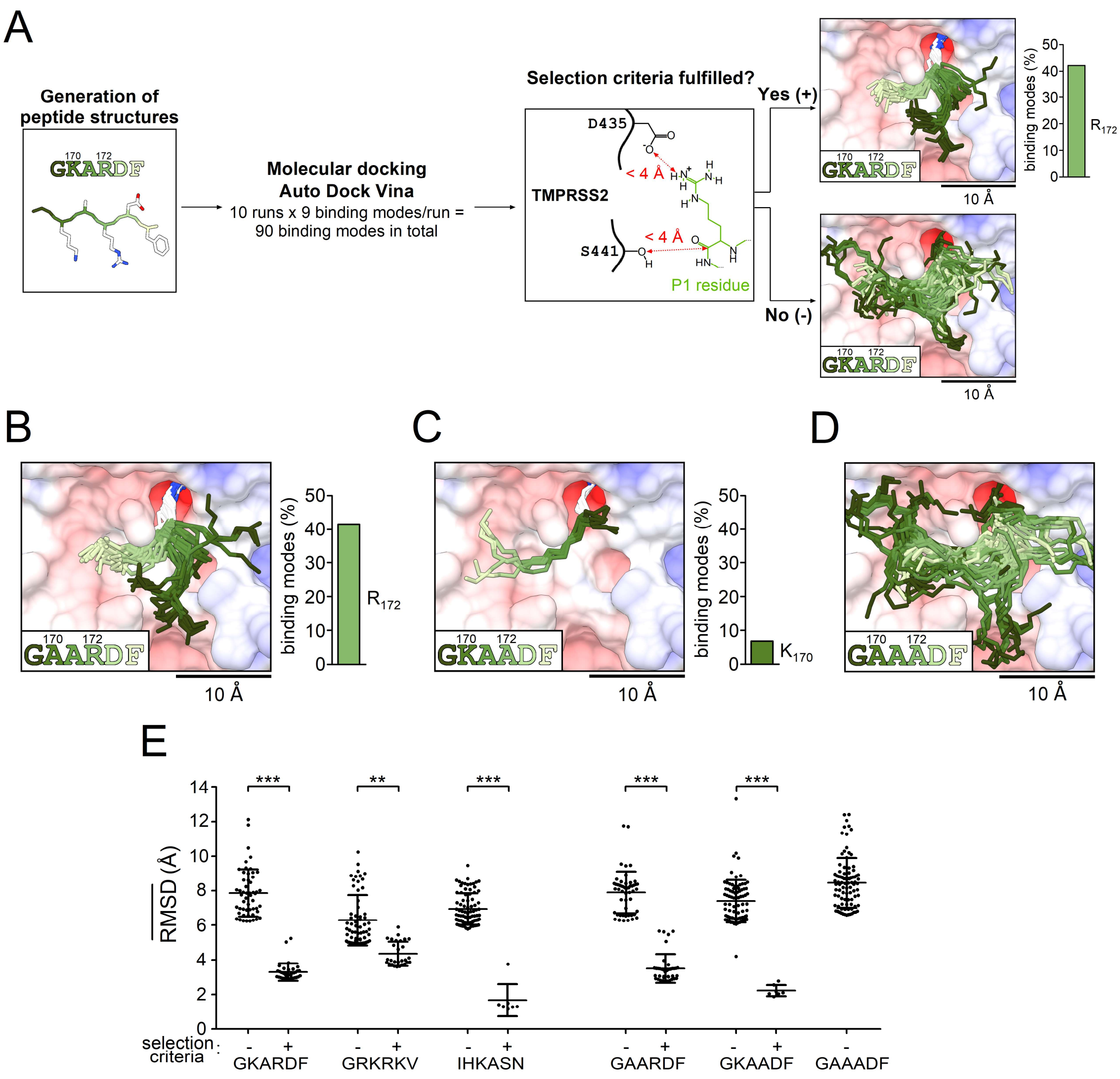

Figure S2. Prediction of putative TMPRSS2 cleavage sites distal to the γ-inhibitory tract using a molecular docking approach. (*A*) Schematic diagram illustrating the molecular docking strategy used to predict binding modes of 6-mer peptides corresponding to different segments of the distal region of the γ-inhibitory tract to the catalytic domain of TMPRSS2 which are compatible with proteolysis, i.e. fulfill the selection criteria. (*B*, *C*) All binding modes that fulfill the selection criteria for the GAARDF- (*B*) and GKAADF-peptide (*C*) are shown. (*D*) All generated binding modes are shown for the GAAADF-peptide, which lacks positively charged arginine or lysine residues. Peptide backbone carbons and nitrogens (in the same color as the corresponding amino acid residue of the peptide sequence given in the lower left corner) and the side chains of arginine or lysine residues occupying the S1 pocket (with carbons in white and nitrogens in blue) are shown. Bar diagrams demonstrate the percentage of the binding modes which fulfill the selection criteria out of the total number of binding modes (90) generated for each peptide, and indicate the arginine or lysine residue that occupies the S1 pocket. (*E*) Binding modes for each simulated peptide were subdivided into groups of binding modes that do not fulfill (−) or fulfill (+) the selection criteria. Average values of the root-mean-square deviation (RMSD) were calculated for each individual binding mode k ($\overline{RMSD_k}$) according to the following equation:

$$\overline{RMSD_k} = \frac{1}{n-1}\sum_{j=1}^{n} RMSD_{kj}$$

where $RMSD_{kj}$ is the RMSD value calculated between all backbone carbon and nitrogen atoms of a peptide binding mode k and the corresponding atoms of a peptide binding mode j ($k \neq j$) from the same group, n is the total number of peptide binding modes in the corresponding group. ***$p<0.001$, **$p<0.01$, Kruskall-Wallis with Dunn's post hoc test ($7 \leq N \leq 90$).

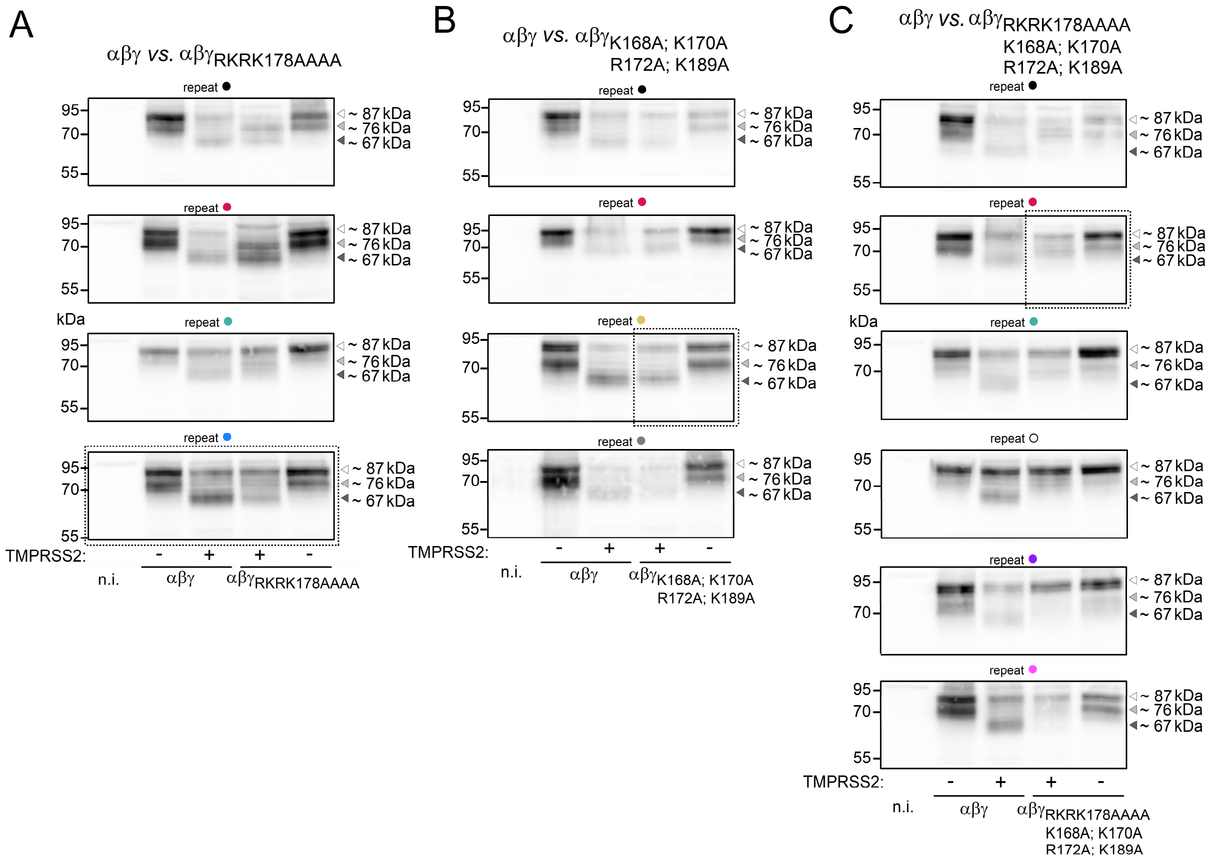

Figure S3. Eliminating putative TMPRSS2 cleavage sites distal to the γ-inhibitory tract prevents the appearance of fully cleaved γ-ENaC in whole-cell lysates. All western blots obtained in similar experiments as shown in Fig. 8C are depicted. Parts of blots shown in Fig. 8C are framed by dotted rectangles. Whole-cell expression of γ-ENaC was detected using an antibody against a C-terminal γ-ENaC epitope in oocytes expressing wild-type (αβγ) or mutant ENaC ($\alpha\beta\gamma_{RKRK178AAAA}$, $\alpha\beta\gamma_{K168A;K170A;R172A;K189A}$ or $\alpha\beta\gamma_{RKRK178AAAA;K168A;K170A;R172A;K189A}$) either without (−) or with (+) TMPRSS2 coexpression. Non-injected oocytes served as control (n.i.). Expression of γ-ENaC was analysed for $\alpha\beta\gamma_{RKRK178AAAA}$- (*A*) and $\alpha\beta\gamma_{K168A;K170A;R172A;K189A}$- (*B*) in four, for $\alpha\beta\gamma_{RKRK178AAAA;K168A;K170A;R172A;K189A}$- (*C*) in six, and for αβγ-ENaC (*A-C*) in nine different repeats indicated by circles of different colours. Positions of uncleaved (~87 kDa), partially cleaved (~76 kDa) and fully cleaved γ-ENaC (~67 kDa) are indicated by open, light grey and dark grey filled arrowheads, respectively.

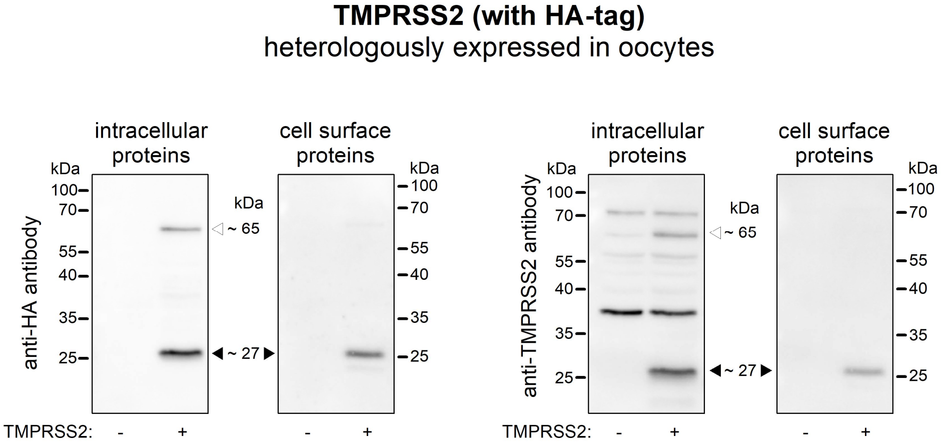

Figure S4. Validation of a TMPRSS2-specific antibody using HA-tagged TMPRSS2 heterologously expressed in *Xenopus laevis* oocytes. Western blot analysis of intracellular and cell surface expression of C-terminally HA-tagged TMPRSS2 in oocytes from one batch was performed as described in Fig. 1E. The blot was first probed with anti-HA antibody (*left two panels*) and then stripped and re-probed using a commercial TMPRSS2-specific antibody (*right two panels*). Importantly, both antibodies specifically detected the mature catalytic chain of TMPRSS2 (~27 kDa band) as indicated by filled arrowheads. The ~3 kDa higher molecular weight observed for the catalytic chain of heterologously expressed TMPRSS2 compared to that of TMPRRS2 endogenously expressed in H441 cells (see Figs. 11A, S8A) corresponds to the expected molecular weight of the HA-tag. The detection of the zymogen form of TMPRSS2 (~65 kDa band, indicated by open arrowheads) by the TMPRSS2-specific antibody was compromised by the presence of a faint non-specific band of similar molecular weight which was also detected in oocytes without TMPRSS2 expression. Nevertheless, these control experiments confirmed the usefulness of the commercial TMPRSS2-specific antibody to detect TMPRSS2 knockdown in H441 human airway epithelial cells.

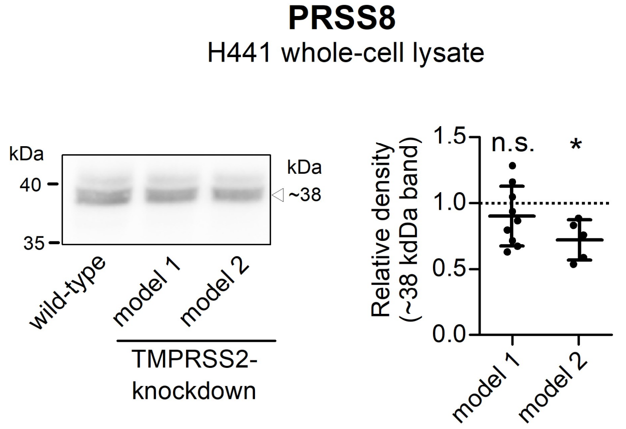

Figure S5. Similar expression of endogenous prostasin (CAP1, PRSS8) in wild-type and TMPRSS2-knockdown H441 cells. *Left panel*, representative western blot showing endogenous whole-cell expression of PRSS8 in H441 cells without (wild-type) or with TMPRSS2-knockdown (model 1 and model 2) detected using a PRSS8-specific antibody. PRSS8 in its activated cleaved form (catalytic heavy chain, ~38 kDa) with hydrolyzed disulfide bonds between the light and heavy chain under reducing WB conditions is indicated by an open arrowhead. *Right panel*, densitometric evaluation of PRSS8 expression from similar blots as shown in *left panel*. In each blot the density value of the ~38 kDa PRSS8 band obtained for TMPRSS2-knockdown model 1 (n=9) or model 2 (n=5) was normalized to that of the corresponding PRSS8 band obtained in wild-type H441 cells. Dashed line indicates the normalized density value of one (no effect). Mean ± SD and individual data points are shown; *$p < 0.05$; n.s., not significant, one sample Student's t test compared to wild-type (1.0).

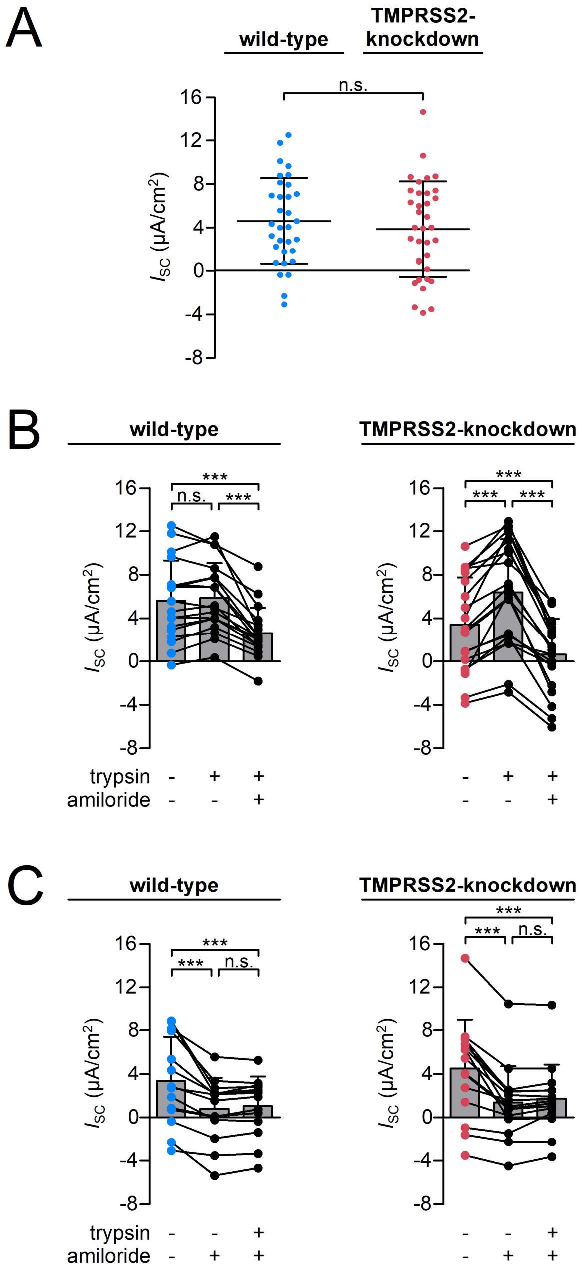

Figure S6. Absolute I_{SC} values obtained in the same experiments as shown in Fig. 12. (*A*) Summary of baseline equivalent short circuit current (I_{SC}) values are shown, which were measured before trypsin (same values as in B) or amiloride (same values as in C) application in wild-type H441 cells (blue points) or TMPRSS2-knockdown H441 cells (model 1, red points). Mean ± SD and individual data points are shown. $32 \leq n \leq 35$; n.s., not significant; two-tailed unpaired Student's *t* test. (*B*, *C*) Absolute I_{SC} values used to calculate $\Delta I_{SC\ trypsin}$ and $\Delta I_{SC\ ami}$ shown in Fig. 12 are depicted. Lines connect data points obtained from an individual H441 cell monolayer. Mean ± SD and individual data points are shown. $14 \leq n \leq 20$; ***$p < 0.001$; n.s., not significant; repeated measures ANOVA with Bonferroni post hoc test.

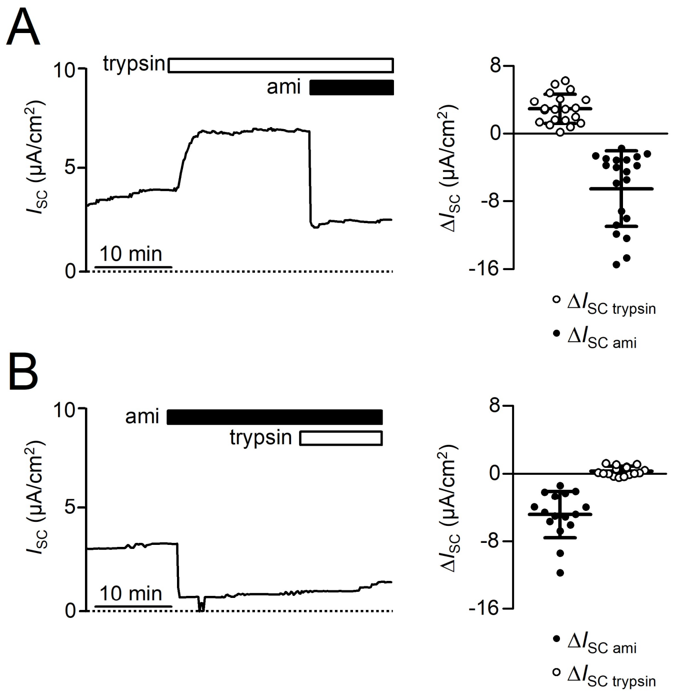

Figure S7. Effect of apical application of trypsin and amiloride on I_{SC} observed in the second TMPRSS2-knockdown H441 cell model (model 2). (*A, B*) Representative equivalent short circuit current (I_{SC}) recordings from TMPRSS2-knockdown H441 cells (model 2) are shown on the left with corresponding summary data from similar experiments shown on the right. Experimental measurements and data analysis were performed as described in Fig. 12. Mean ± SD and individual data points are shown ($16 \leq n \leq 20$).

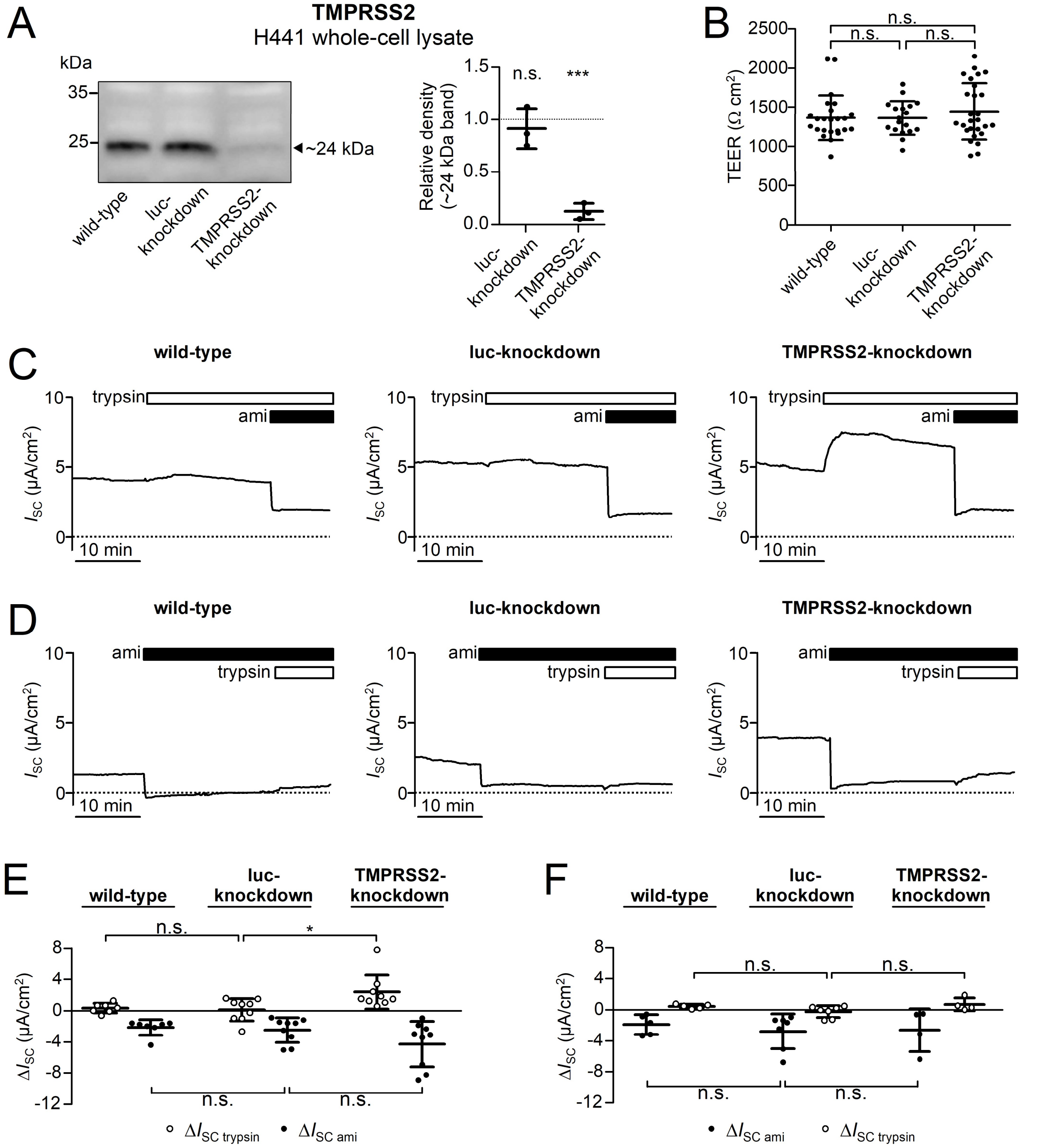

Figure S8. Properties of luc-knockdown H441 cells are similar to those of wild-type H441 cells but differ from those of TMPRSS2-knockdown cells. TMPRSS2 protein expression detection (*A*, n=3) and transepithelial electrical resistance (TEER) measurements (*B*, $18 \leq n \leq 27$) were performed and analyzed as described in Fig. 11. Equivalent short circuit current (I_{SC}) recordings (*C-F*; $4 \leq n \leq 9$) were performed and analyzed as described in Fig. 12. E and F summarize data from experiments similar to those shown in C and D, respectively. Data points from wild-type and TMPRSS2-knockdown H441 cells (model 1) shown in E and F are also included in the summary data shown in Figs. 11, 12 and S6.
